# Supplementary material for: Imbalance of stem-like and effector T cell states in children with early type 1 diabetes across conventional and regulatory subsets
Source: Nat Commun. 2025 Dec 11;16:11301. doi: 10.1038/s41467-025-66459-4 (PMC12722300; doi:10.1038/s41467-025-66459-4)
Supplement: Supplementary file 1 — Supplementary Information [file 41467_2025_66459_MOESM1_ESM.pdf]

Supplementary Information: Imbalance of stem-like and effector T cell states in children with early type 1 diabetes across conventional and regulatory subsets

Veronika Niederlova[1,2], Ales Neuwirth[1], Vit Neuman[3], Juraj Michalik[1], Bela Charvatova[1], Martin Modrak[4], Zdenek Sumnik[3], Ondrej Stepanek[1,*]

1 Laboratory of Adaptive Immunity, Institute of Molecular Genetics of the Czech Academy of Sciences, Prague, Czechia

2 Department of Cell Biology, Faculty of Science, Charles University in Prague, Czechia

3 Department of Pediatrics, 2nd Faculty of Medicine, Charles University & Motol University Hospital, Prague, Czechia

4 Department of Bioinformatics, 2nd Faculty of Medicine, Charles University, Prague, Czechia

* Correspondence should be addressed to Ondrej Stepanek, ondrej.stepanek@img.cas.cz

This Supplementary Information file contains:

- Supplementary Figures 1-22
- Supplementary Tables 1-7
- References to Supplementary Information

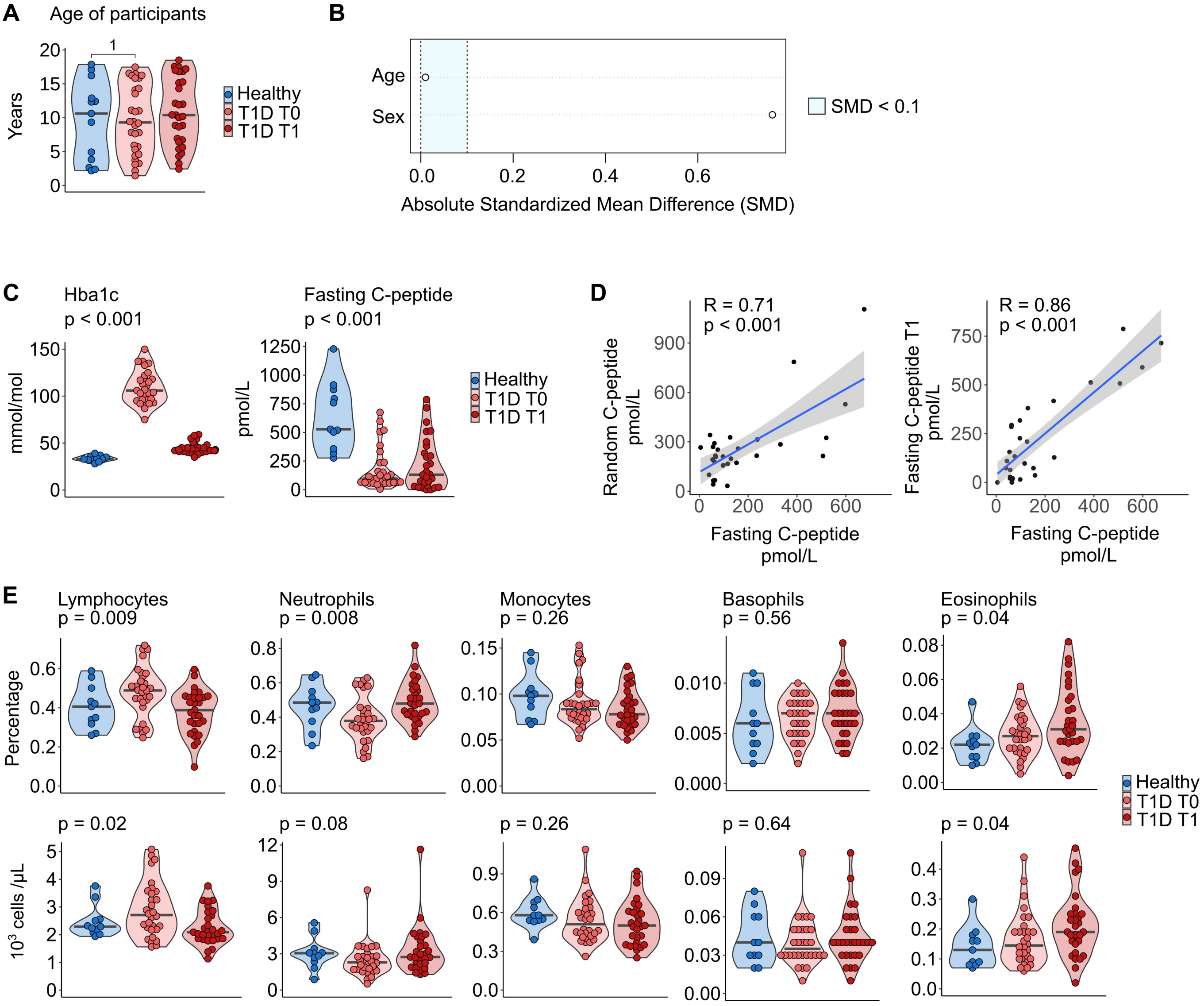

Supplementary Figure 1. Peripheral blood profiling in healthy children and type 1 diabetes at diagnosis and follow-up. Peripheral blood from fasting blood sampling was analyzed by routine clinical testing. **(A)** Violin plots showing the age of healthy donors, children with T1D at diagnosis and the same patients at one-year follow-up. P-value was calculated using the two-tailed Mann-Whitney test between healthy donors and T1D T0. Bars at median. n = 13 Healthy, n = 30 T1D T0, n = 29 T1D T1. **(B)** Standardized mean difference analysis of the distribution of age and sex in healthy donors and children with T1D calculated by the MatchIt package. SMD < 0.1 indicated balanced groups. **(C)** Levels of glycosylated hemoglobin (left) and C-peptide (right) in fasting blood samples of healthy donors, children with T1D at diagnosis and the same patients at one-year follow-up. P-value was calculated using the Kruskal-Wallis test. Bar at median. n = 11 Healthy, n = 29 T1D T0, n = 28 T1D T1. **(D)** Correlation of the levels of fasting C-peptide from blood samples of T1D patients at T0 and the levels of C-peptide sampled at a random timepoint (left), or with the levels of fasting C-peptide sampled from the same patients at T1 (right). P-value was calculated using the Pearson's correlation test. n = 28 T1D donors. Line = regression fit; shaded area = 95% CI. **(E)** Percentages (upper panels) and absolute counts (lower panels) of major white blood cell populations of healthy donors and T1D at T0 and T1. P-value was calculated using the Kruskal-Wallis test. Bar at median. n = 11 Healthy, n = 29 T1D T0, n = 28 T1D T1.

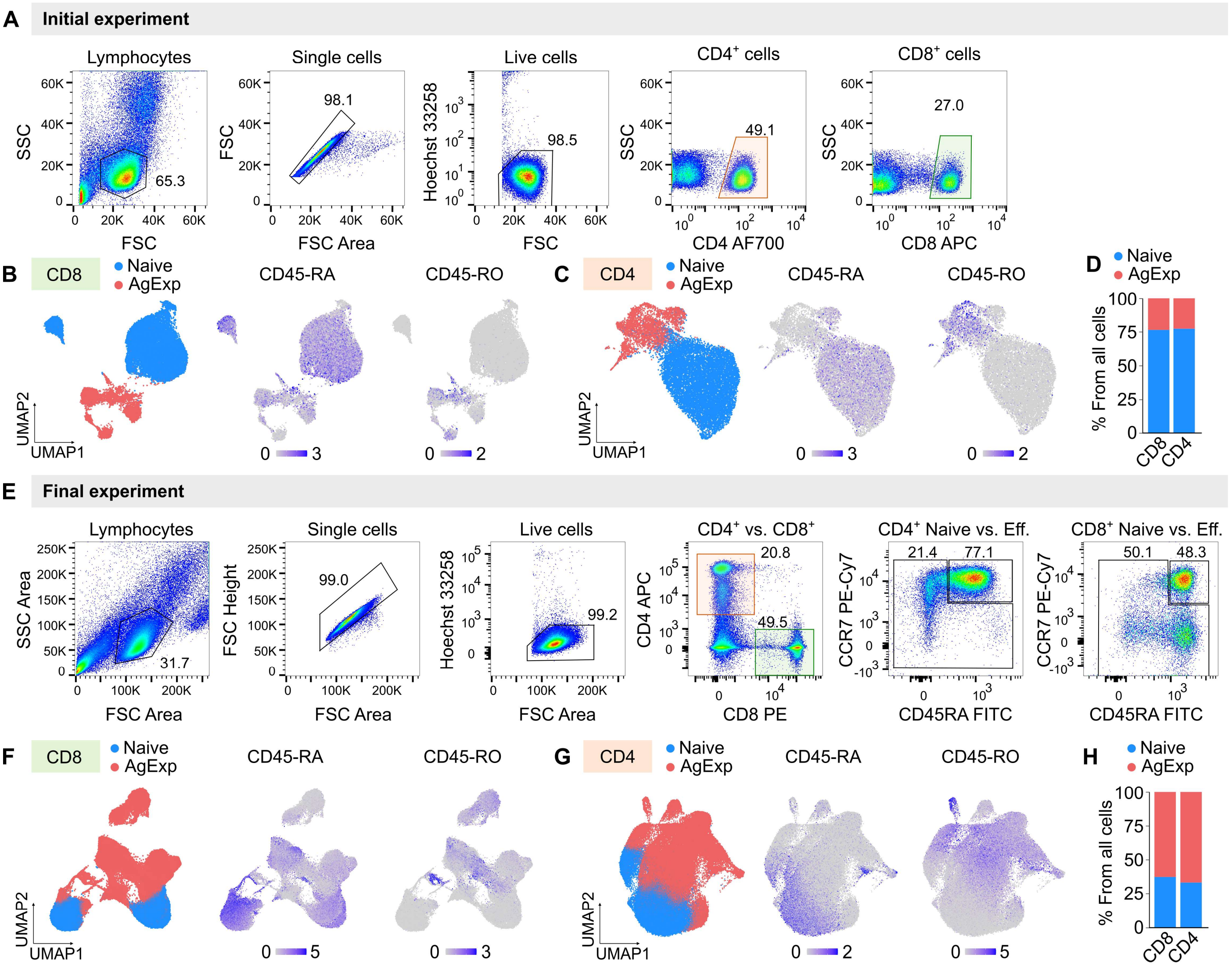

Supplementary Figure 2. Experimental design in initial and final experiments. (A-D) An initial experiment in which CD4+ and CD8+ T cells were sorted from 12 T1D children and 6 healthy donors. **(A)** Representative gating used for sorting of CD4+ and CD8+ T cells from fresh frozen PBMCs. **(B)** UMAP plot showing CD8+ T cells from initial experiments before quality control and removal of contaminating cells. Clusters of cells were annotated as naïve (blue) or antigen-experienced (AgExp, red) based on marker genes and isoforms of CD45 (PTPRC) inferred from gene expression data. n = 18,270 cells from 18 donors. **(C)** Same as B, but for CD4+ T cells. n = 14,278 cells from 18 donors. **(D)** Quantification of naïve and AgExp clusters in the initial CD4+ and CD8+ T-cell datasets. **(E-H)** Final experiment in which AgExp enriched CD4+ and CD8+ T cells were sorted from 30 T1D donors and 13 healthy donors. For each patient, naïve and AgExp cells were sorted at 1:5 ratio. **(E)** Representative gating used for sorting of AgExp enriched CD4+ and CD8+ T cells from fresh frozen PBMCs. **(F)** The UMAP plot shows CD8+ T cells from final experiments before quality control and removal of contaminating cells. Clusters of cells were annotated as naïve (blue) or AgExp (red) based on marker genes and isoforms of PTPRC inferred from gene expression data. n = 93,412 cells from 43 donors. **(G)** Same as F but for CD4+ T cells. n = 78,635 cells from 43 donors. **(H)** Quantification of naïve and AgExp clusters in the final CD4+ and CD8+ T-cell datasets.

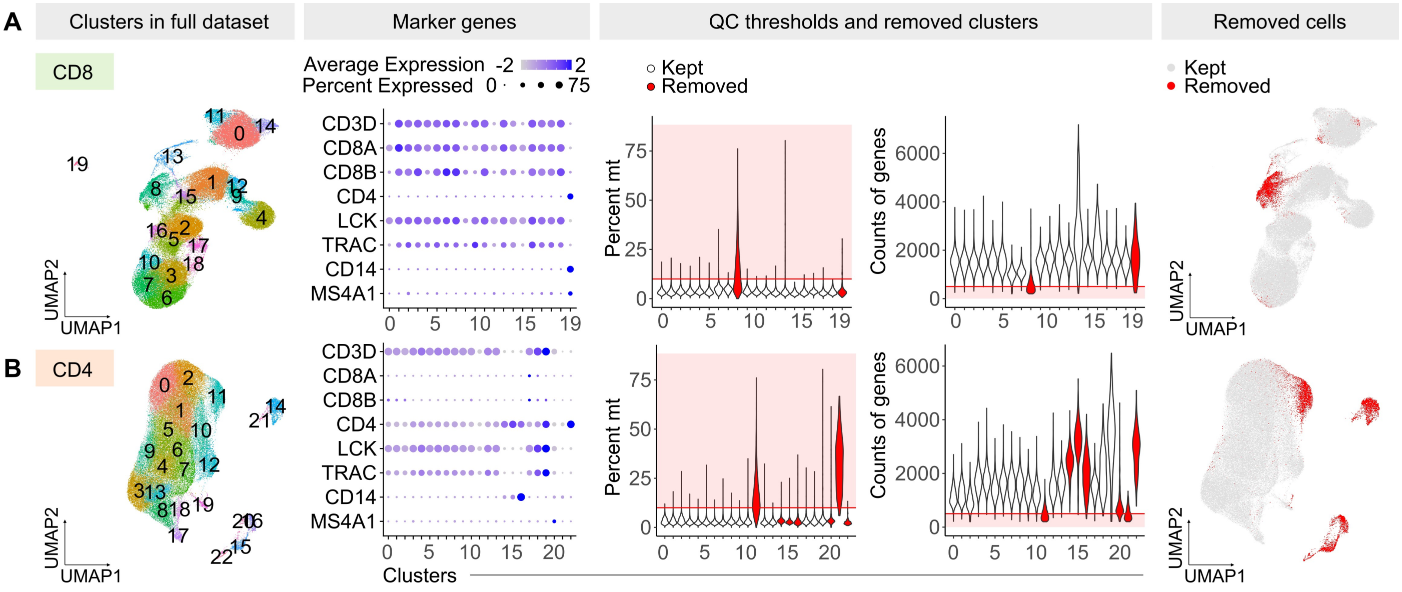

Supplementary Figure 3. Quality control of T-cell datasets. (A-B) The quality control and filtering of cells from the initial and final experiments in the CD8+ T-cell dataset **(A)** and CD4+ T-cell dataset **(B)**. From left are shown: the UMAP plots of all cells before quality control with Louvain clusters; DotPlots showing the expression of canonical T-cell genes and genes characteristic for contaminating cell types in the clusters; Violin plots showing percentages of mitochondrial genes and total gene counts in all clusters together with filtering criteria (red = removed, white = kept); and UMAP plots showing the cells that passed the quality control (grey) or were removed (red).

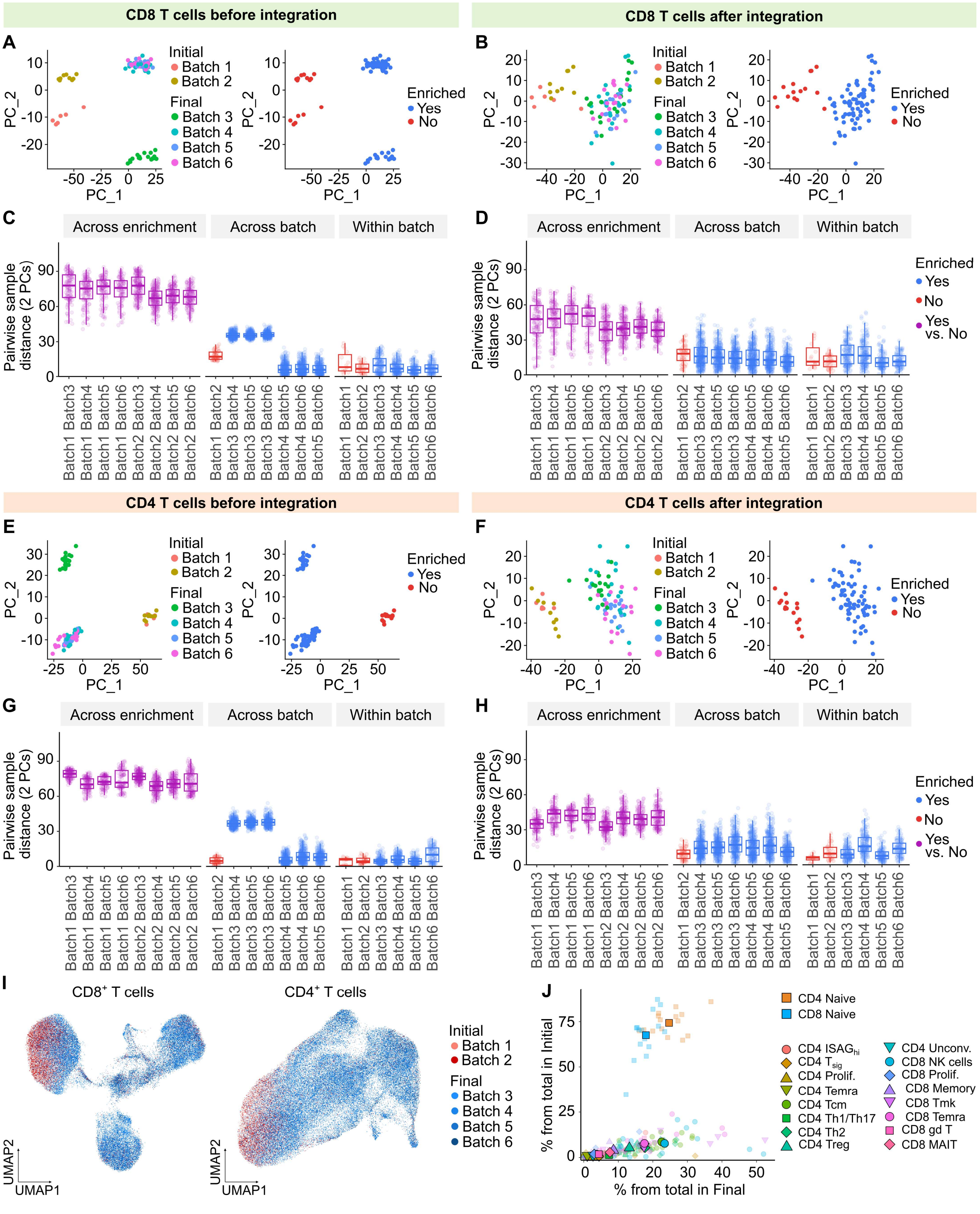

Supplementary Figure 4. Evaluation of dataset integration. (A) Sample-level PCA analysis of CD8+ samples before integration. Each dot represents one sample. Samples are colored by batch (left) and enrichment (right). **(B)** Same as (A), but after integration with STACAS. **(C)** Quantification of distances between CD8+ samples in the two-dimensional PCA space shown in (A) before integration. Colors represent different comparisons: across enrichment (expected biological variability due to different enrichment strategies), across batch (unwanted technical variability), and within batch (variability between individual donors). **(D)** Quantification of distances between CD8+ samples in the two-dimensional PCA space shown in (B) after integration. Color code as in (C). **(E)** Same as A), but for CD4+ T cells. **(F)** Same as B), but for CD4+ T cells. **(G)** Same as C), but for CD4+ T cells. **(H)** Same as D), but for CD4+ T cells. **(I)** The same projections as in Fig. 1C (CD8+ T cells) and Fig. 1F (CD4+ T cells). Cells are colored by batch. **(J)** Dot plot showing the proportions of cell populations in the same sample analyzed twice. Each sample was analyzed both in the initial experiment (without enrichment) and in the final experiment, in which non-naïve populations were enriched at a 1:5 naïve:non-naïve ratio. Each semitransparent data point represents one population from one sample. Larger full-color data points of the same shape and color represent the centroid of all samples from that population.

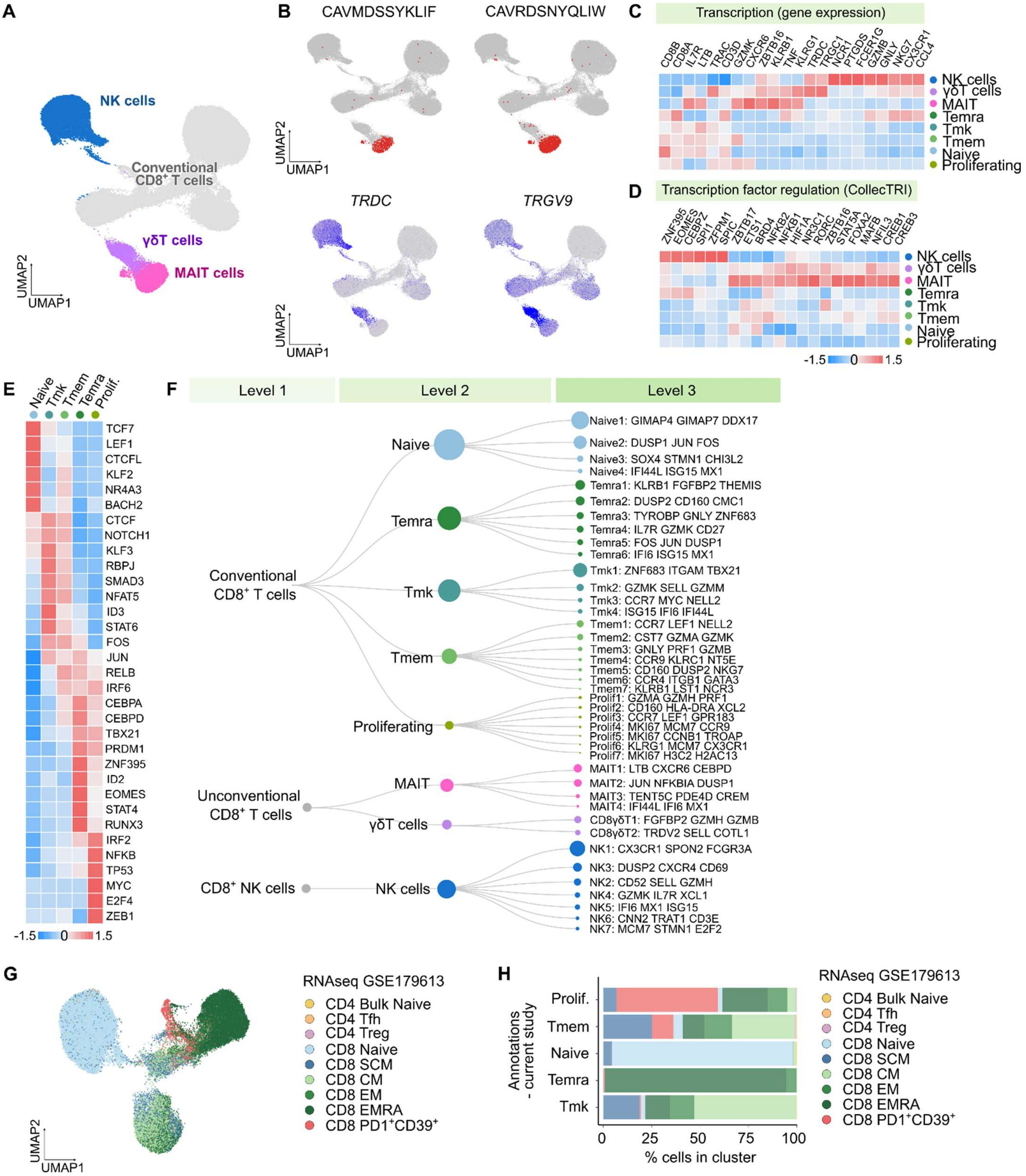

Supplementary Figure 5. Analysis of CD8+ T cells. (A) The UMAP projection of CD8+ T cells showing the unconventional subsets and NK cells, which were not included in the main analysis shown in Fig. 1C-E. Louvain clusters were merged based on the functional relevance. n = 95,229 cells from 43 donors. **(B)** The same UMAP projection as in (A) showing the detection of TCR CDR3α sequences typical for MAIT cells [1] in the top panels and the expression of TRDC and TRGV9 gene segments in the bottom panels. **(C)** The heatmap shows the relative expression of the marker genes that characterize clusters presented in (A). For comparison, clusters of conventional CD8+ T cells (Fig. 1C) were also included. Colors represent row-scaled z-score of average expression of a gene in a cluster. **(D)** The heatmap shows the transcriptional regulation of clusters presented in (A). The transcription factors were identified as the key regulators based on differential expression in each cluster using the CollecTRI transcriptional regulons database [2]. For comparison, clusters of conventional CD8+ T cells (Fig. 1C) were also included. **(E)** The heatmap shows the transcriptional regulation of clusters presented in Fig. 1C. The transcription factors were identified as the key regulators based on differential expression in each cluster using CollecTRI transcriptional regulons database [2]. **(F)** The Sankey plot shows the CD8+ T-cell populations (Level 1) and their subclustering into the main clusters (Level 2) and subclusters (Level 3). Sizes of the dots represent the relative abundance of the cluster. **(G)** Same UMAP projection as in Fig. 1C. Cells are colored by annotations with a reference RNAseq dataset GSE179613. **(H)** Quantification of annotations from reference RNAseq dataset GSE179613 in clusters from Fig. 1C.

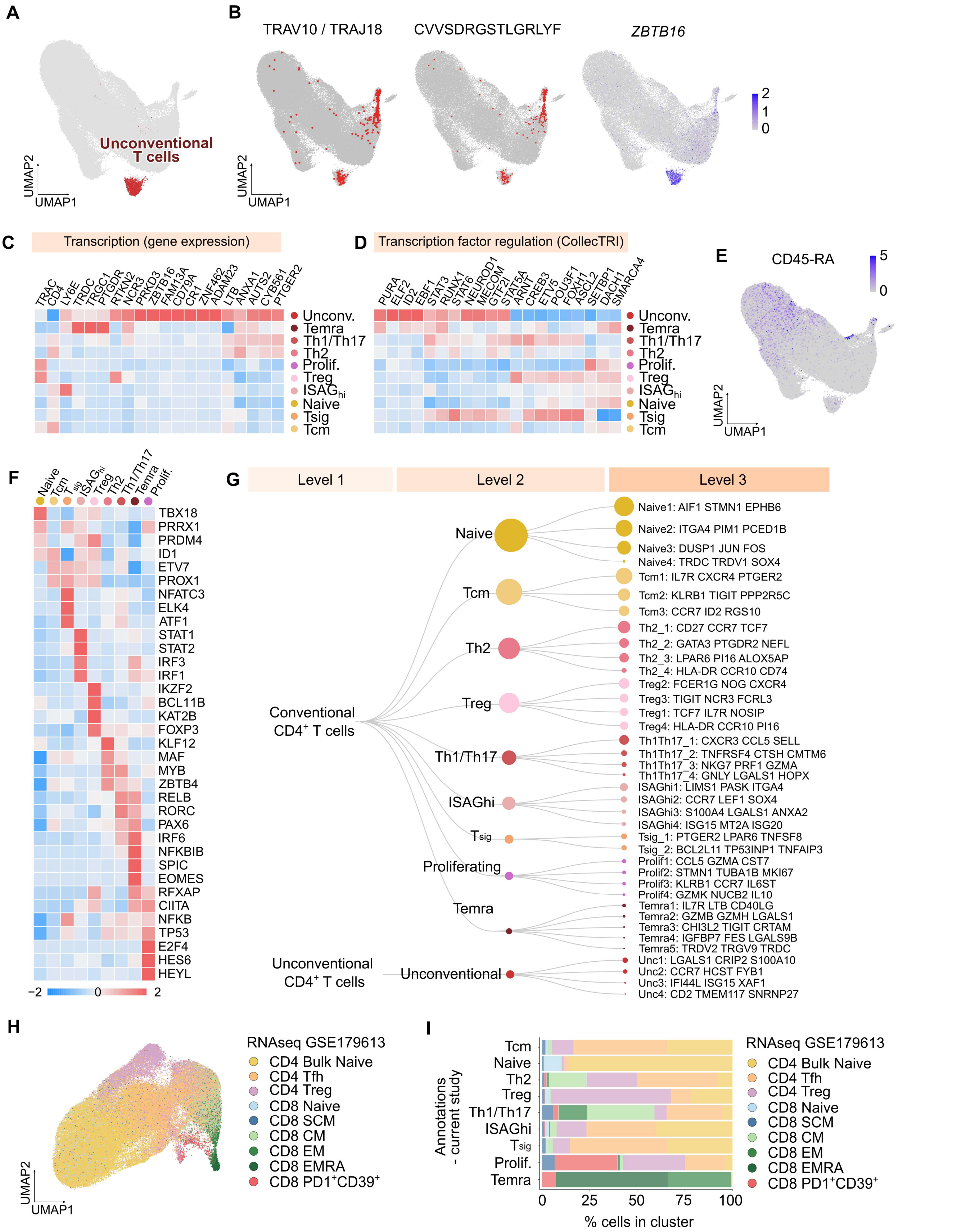

Supplementary Figure 6. Analysis of CD4+ datasets. (A) The UMAP projection of CD4+ T cells showing the unconventional cells. n = 79,876 cells from 43 donors. **(B)** The same UMAP projection as in (A) showing the detection of TRA gene segments TRAV10 recombined with TRAJ18 (left), and the CDR3α sequence typical for iNKT cells [1] (middle). The expression of ZBTB16 is shown (right). **(C)** The heatmap shows the relative expression of marker genes that characterize clusters presented in (A). For comparison, clusters of conventional CD4+ T cells (Fig. 1F) were also included. Colors represent row-scaled z-score of average expression of a gene in a cluster. **(D)** The heatmap shows the transcriptional regulation of the unconventional cells in comparison to conventional clusters. Transcription factors were identified as the key regulators based on differential expression in each cluster using the CollecTRI transcriptional regulons database [2]. **(E)** Identification of reads mapping specifically to CD45-RA (PTPRC isoform RA) by the IDEIS software [3]. **(F)** Same as D), but for clusters presented in Fig. 1F. **(G)** The Sankey plot shows the CD4+ populations (Level 1) and their subclustering into the main clusters (Level 2) and subclusters (Level 3). Sizes of the dots represent relative abundance of the cluster. **(H)** Same UMAP projection as in Fig. 1F. Cells are colored by annotations with a reference RNAseq dataset GSE179613. **(I)** Quantification of annotations from reference RNAseq dataset GSE179613 in clusters from Fig. 1F.

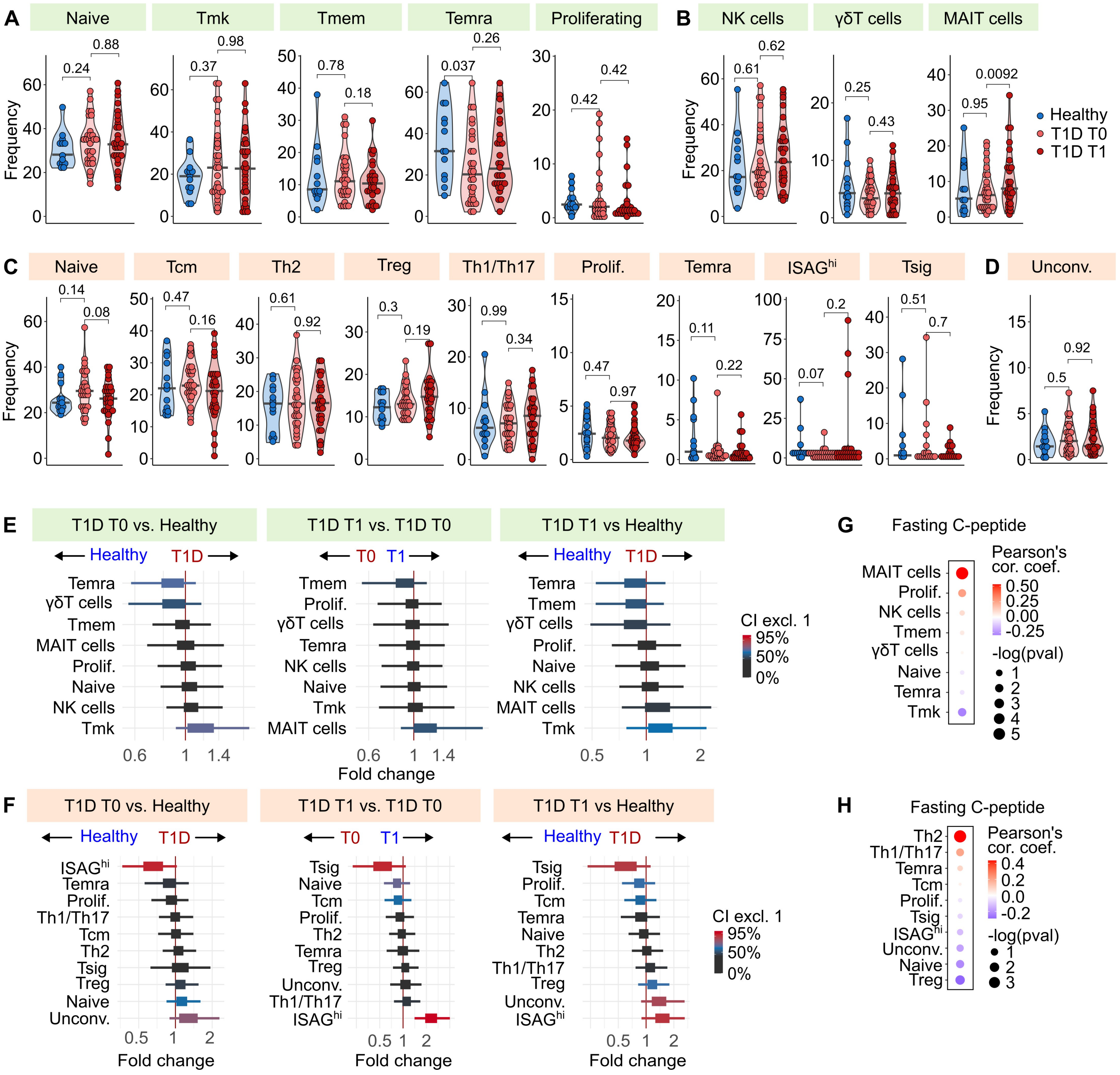

Supplementary Figure 7. Quantification of cluster abundance. (A) Quantification of the cluster composition for clusters shown in Fig. 1C. The violin plots show the percentage of cells in each subcluster from total conventional CD8+ T cells in healthy and T1D donors at T0 and T1. **(B)** Quantification of the cluster composition for clusters shown in Fig. S3A. The violin plots show the percentage of cells in each subcluster from total CD8+ T cells in healthy and T1D donors at T0 and T1. **(C)** The quantification of the cluster composition for clusters shown in Fig. 1F. The violin plots show the percentage of cells in each subcluster from total conventional CD4+ T cells in healthy and T1D donors at T0 and T1. **(D)** The quantification of the unconventional CD4+ cells shown in Fig. S4A. Violin plots show the percentage of unconventional CD4+ T cells from total CD4+ T cells in healthy and T1D donors at T0 and T1. **(E)** The Bayesian analysis of the abundance of subsets of CD8+ T cells in T1D donors at T0 vs. healthy donors (left), T1D donors at T1 vs. T0 (middle) and T1D donors at T1 vs. healthy donors. **(F)** The Bayesian analysis of the abundance of subsets of CD4+ T cells in T1D donors at T0 vs. healthy donors (left), T1D donors at T1 vs. T0 (middle) and T1D donors at T1 vs. healthy donors. **(G)** Correlation of the levels of fasting C-peptide from T1D donors at T1 with the frequency of their populations of CD8+ T cells at T0. **(H)** Correlation of the levels of fasting C-peptide from T1D donors at T1 with the frequency of their populations of CD4+ T cells at T0. A-D - P-value was calculated using two-tailed Mann-Whitney test between healthy donors and T1D T0, or two-tailed paired Mann-Whitney test between T1D donors at T0 and T1. Bars at median.

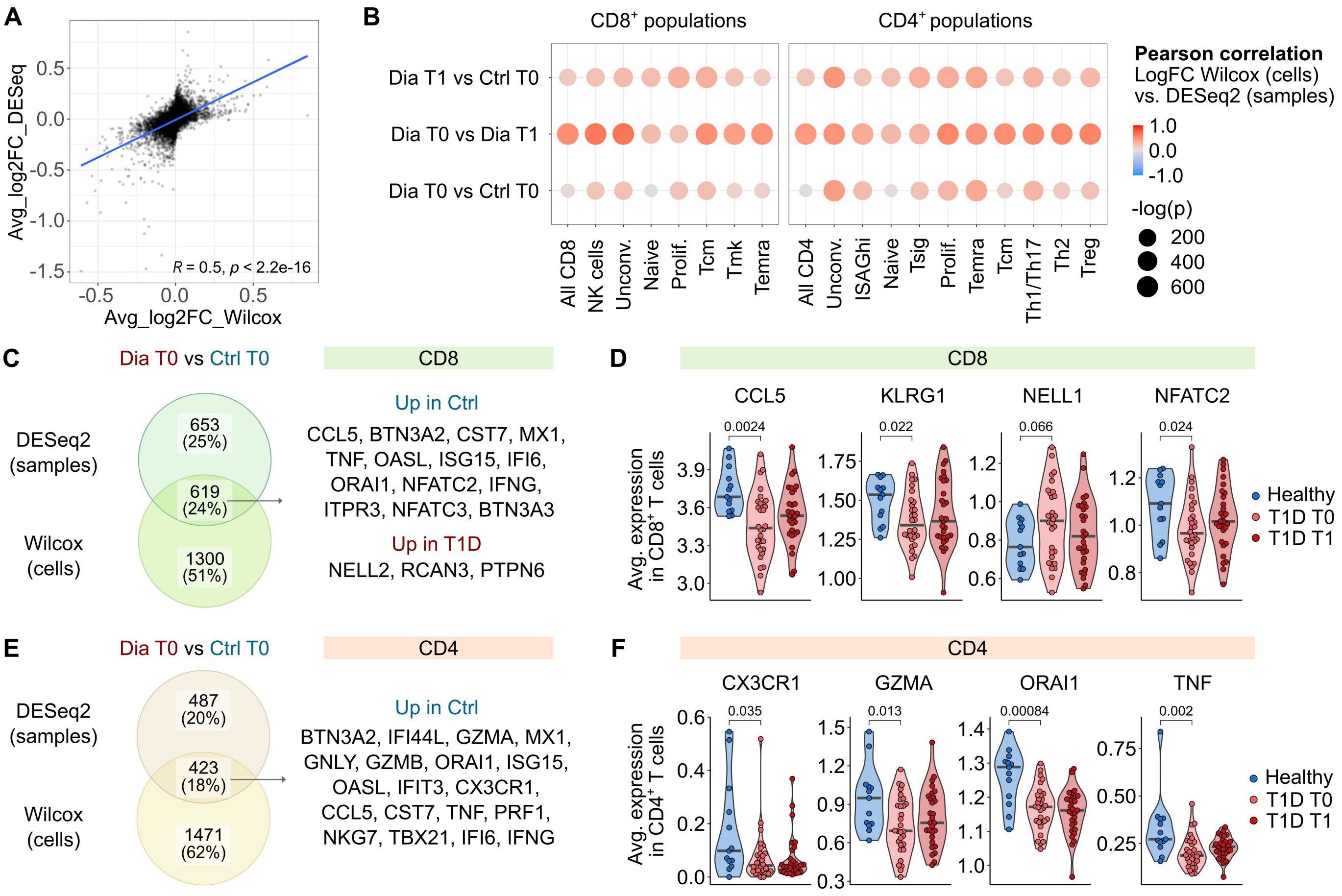

Supplementary Figure 8. Pseudobulk analysis by DESeq2. (A) Representative plot showing the correlation of average log2 fold changes obtained from testing differential expression between pseudobulked T1D CD8+ samples at T0 and T1 using sample-level analysis with DESeq2 (y axis) and using cell-level analysis using Wilcoxon test (default method in the Seurat package). Each dot represents one gene. P-value was calculated using Pearson's test. Line = regression fit. **(B)** Dot plot showing the correlation coefficients and p-values between log2 fold changes obtained from sample-level analysis with DESeq2 (y axis) and cell-level analysis using Wilcoxon test. Representative analysis for one contrast and one population is shown in (A). **(C)** Venn diagram showing the overlap between DEG identified in CD8+ T cells by sample-level analysis with DESeq2 (y axis) and using cell-level analysis using Wilcoxon test (default method in the Seurat package). Shown are genes from the overlapping region which were also present in one of the signatures in Fig 2B. **(D)** Violin plots showing the expression of selected genes in pseudobulked samples of CD8+ T cells. Each dot represents one sample. Bars at median. n = 13 Healthy, n = 30 T1D T0, n = 29 T1D T1. P-value was calculated using two-tailed Mann-Whitney test between healthy donors and T1D T0, or two-tailed paired Mann-Whitney test between T1D donors at T0 and T1. **(E)** Venn diagram showing the overlap between DEG identified in CD4+ T cells by sample-level analysis with DESeq2 (y axis) and using cell-level analysis using Wilcoxon test (default method in the Seurat package). Shown are genes from the overlapping region which were also present in one of the signatures in Fig 2B. **(F)** Violin plots showing the expression of selected genes in pseudobulked samples of CD4+ T cells. Each dot represents one sample. Bars at median. Sample counts and p-values as in (D).

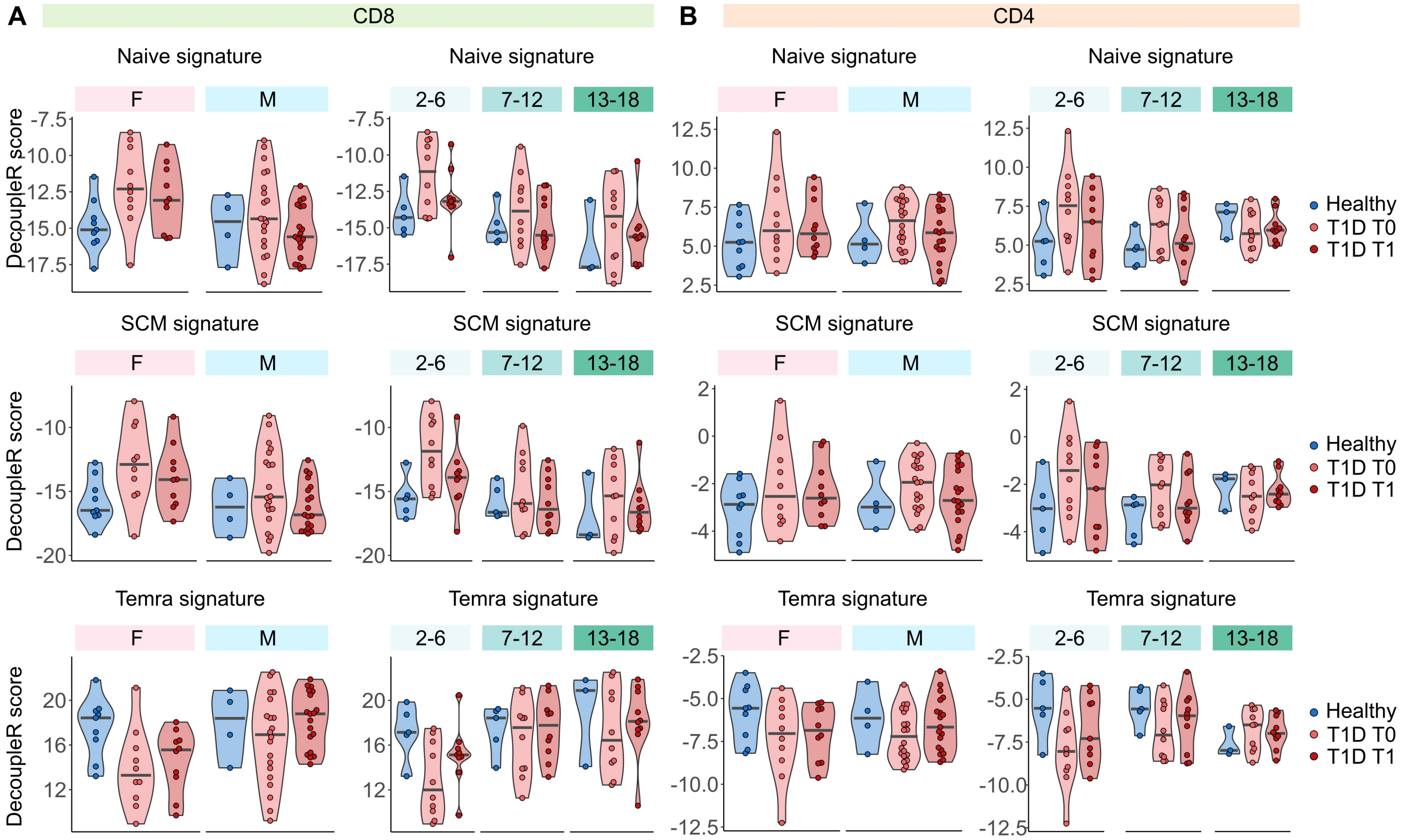

Supplementary Figure 9. Analysis of Naïve, SCM and Temra signatures in sex and age groups. (A-B) Violin plots showing decoupleR scores quantifying similarity to reference gene expression contrasts (Temra signature: Temra vs. naïve, Naïve signature: Naïve vs. Tem/Tcm, Stem-cell memory (SCM) signature: SCM vs. Tem/Tcm from dataset GSE179613 [4]) in CD8+ T cells (A) and CD4+ T cells (B). Each dot represents an individual sample. Data were disaggregated by sex (left) or age (right) of donors. Bars indicate medians.

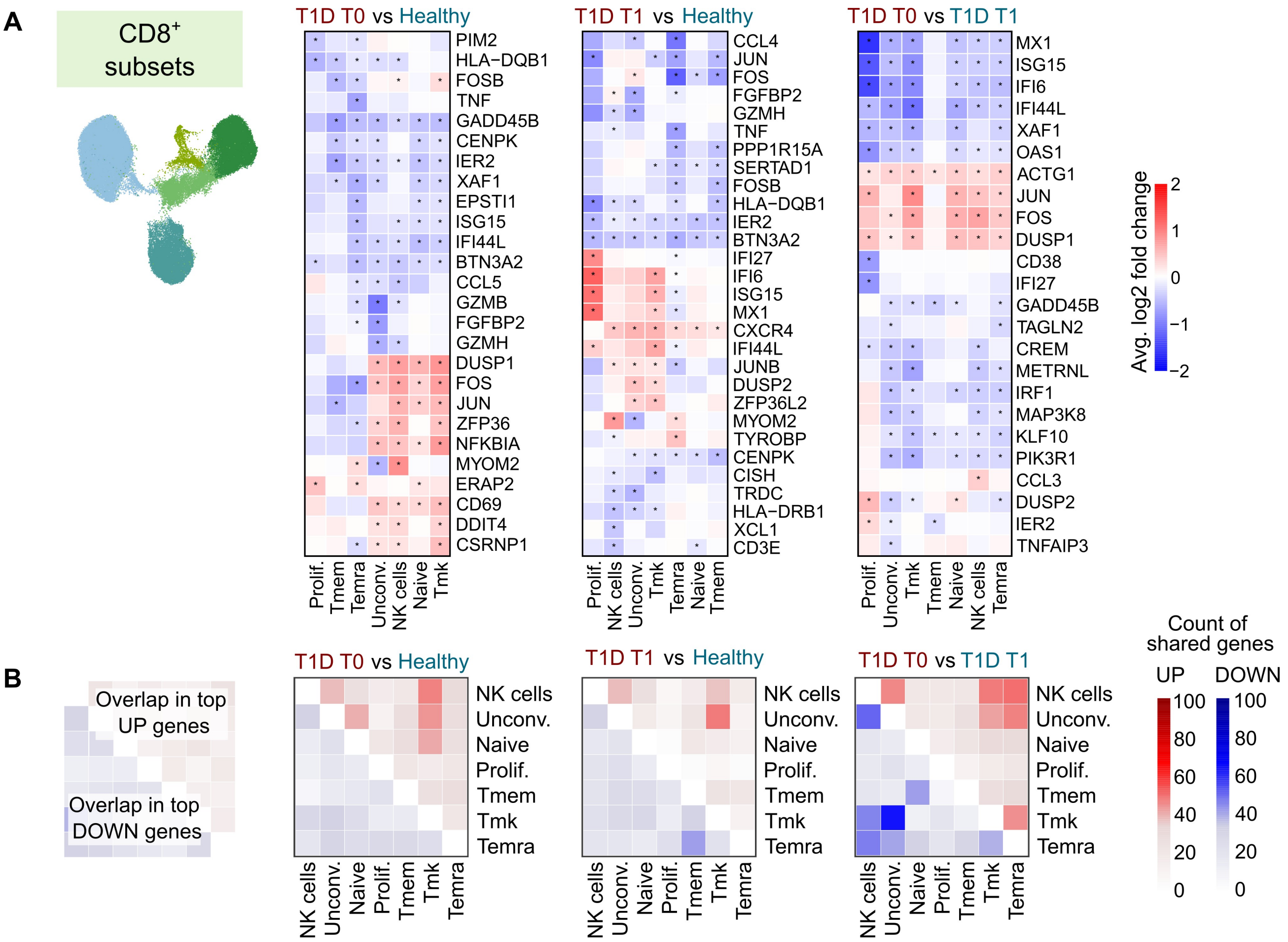

Supplementary Figure 10. Analysis of DEG in subpopulations of CD8+ T cells. (A) Differentially expressed genes in subpopulations of CD8+ T cells between T1D at T0 vs. healthy donors (left), T1D at T1 vs. healthy donors (middle) and T1D at T0 vs. T1D T1 (right). The genes that are shown were selected as the union of the top5 DEG for each subpopulation. Color indicates the avg. log2FC from using cell-level analysis using Wilcoxon test (default method in the Seurat package). Stars indicate statistically significant differences after Bonferroni adjustment for multiple comparisons. **(B)** Heatmaps showing the overlap of the top 100 upregulated (red color) or top 100 downregulated (blue color) genes between the subpopulations of CD8+ T cells. The top 100 genes were the 100 genes with the highest or lowest log2 FC obtained from the following contrasts: T1D at T0 vs. healthy donors (left), T1D at T1 vs. healthy donors (middle) and T1D at T0 vs. T1D T1 (right) using cell-level analysis using Wilcoxon test (default method in the Seurat package).

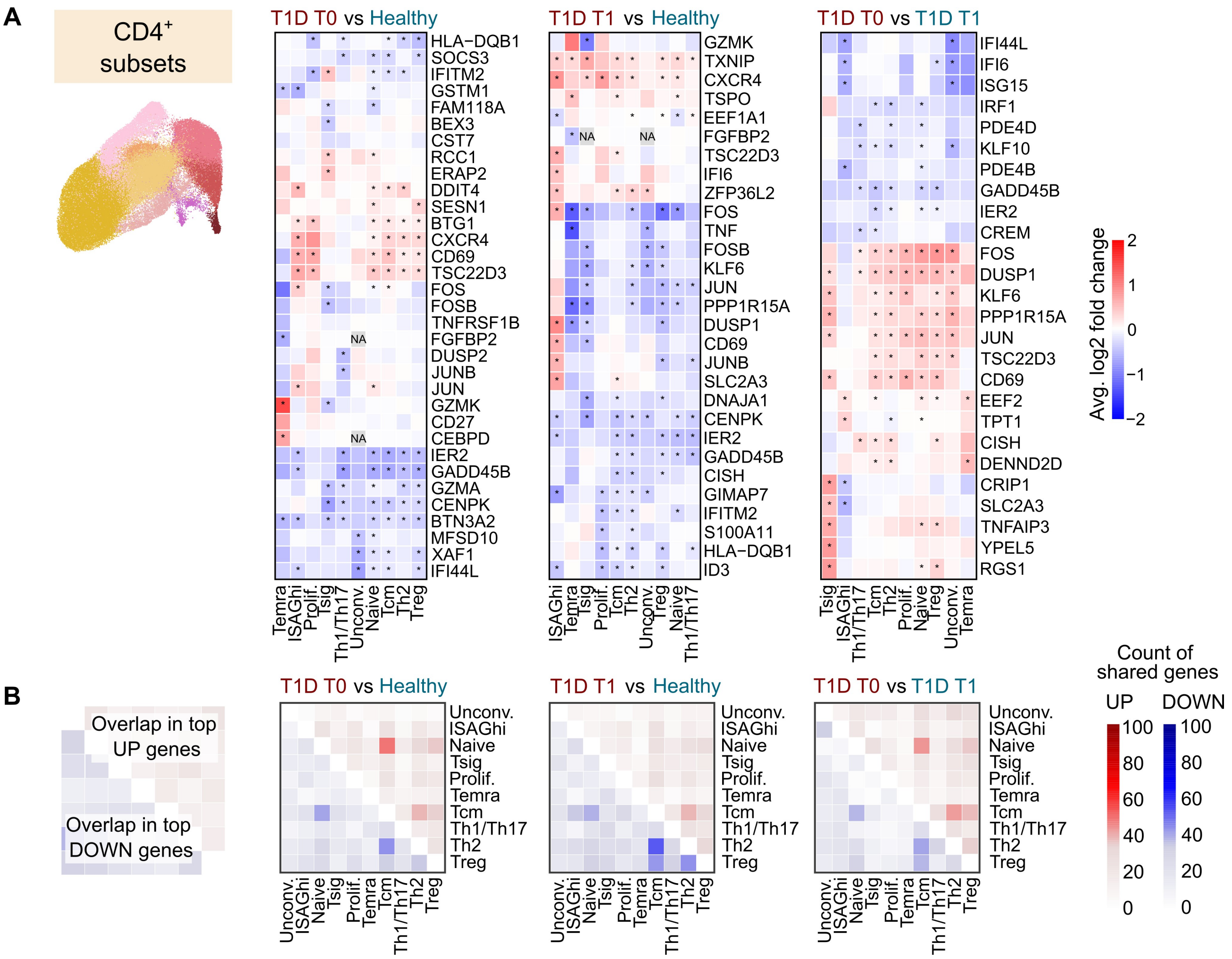

Supplementary Figure 11. Analysis of DEG in subpopulations of CD4+ T cells. (A) Differentially expressed genes in subpopulations of CD4+ T cells between T1D at T0 vs. healthy donors (left), T1D at T1 vs. healthy donors (middle) and T1D at T0 vs. T1D T1 (right). The genes that are shown were selected as the union of the top5 DEG for each subpopulation. Color indicates the avg. log2FC from using cell-level analysis using Wilcoxon test (default method in the Seurat package). Stars indicate statistically significant differences after Bonferroni adjustment for multiple comparisons. **(B)** Heatmaps showing the overlap of the top 100 upregulated (red color) or top 100 downregulated (blue color) genes between the subpopulations of CD4+ T cells. The top 100 genes were the 100 genes with the highest or lowest log2 FC obtained from the following contrasts: T1D at T0 vs. healthy donors (left), T1D at T1 vs. healthy donors (middle) and T1D at T0 vs. T1D T1 (right) using cell-level analysis using Wilcoxon test (default method in the Seurat package).

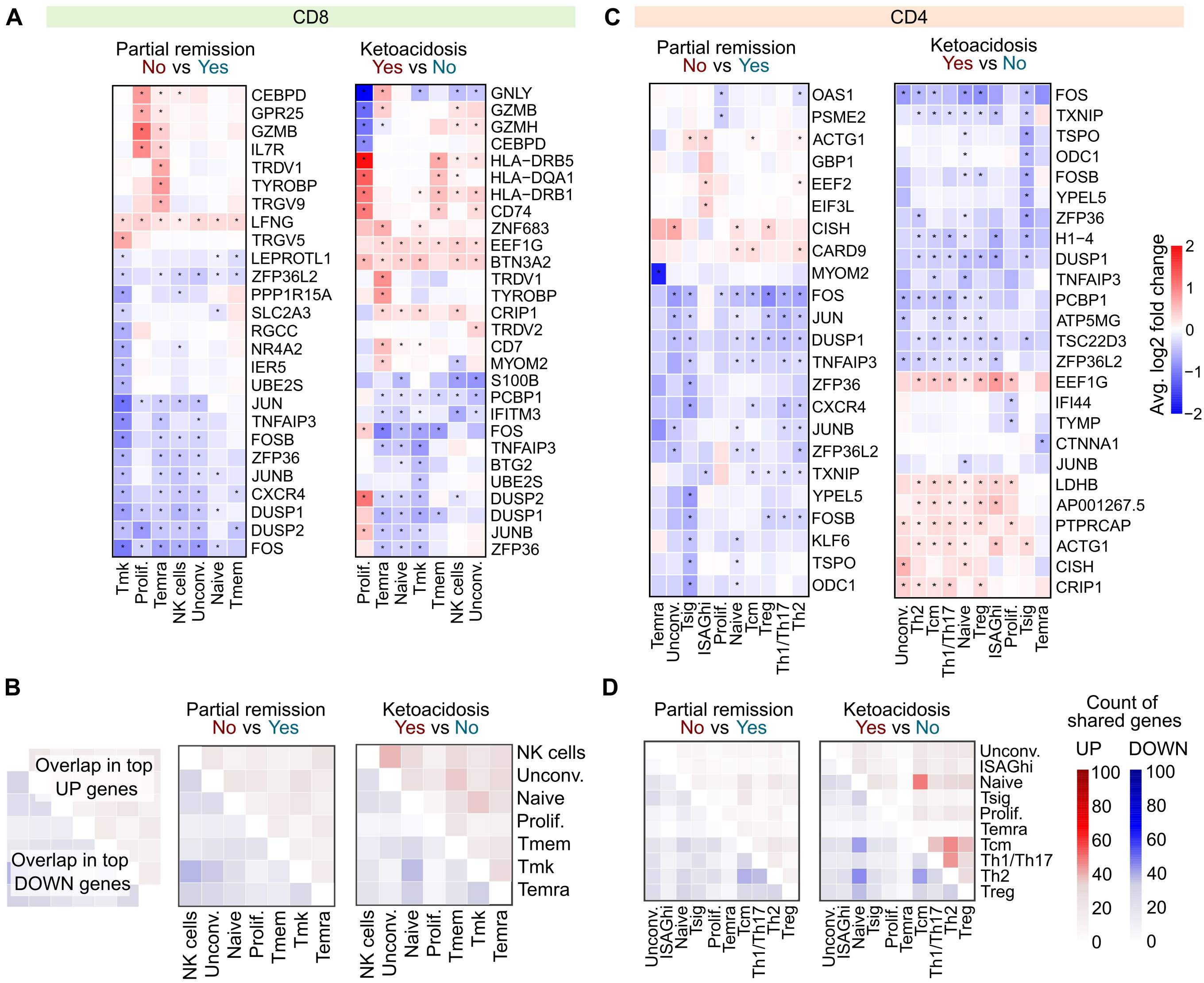

Supplementary Figure 12. Analysis of DEG in ketoacidosis and partial remission. (A) Differentially expressed genes in subpopulations of CD8+ T cells between T1D donors without partial remission vs. with partial remission (left), and T1D donors with ketoacidosis vs. without ketoacidosis (right). The genes that are shown were selected as the union of the top5 DEG for each subpopulation. Color indicates the avg. log2FC from using cell-level analysis using Wilcoxon test (default method in the Seurat package). Stars indicate statistically significant differences after Bonferroni adjustment for multiple comparisons. **(B)** Heatmaps showing the overlap of the top 100 upregulated (red color) or top 100 downregulated (blue color) genes between the subpopulations of CD8+ T cells. The top 100 genes were the 100 genes with the highest or lowest log2 FC obtained from the following contrasts: T1D donors without partial remission vs. with partial remission (left), and T1D donors with ketoacidosis vs. without ketoacidosis (right) using cell-level analysis using Wilcoxon test (default method in the Seurat package). **(C)** Differentially expressed genes in subpopulations of CD4+ T cells between T1D donors without partial remission vs. with partial remission (left), and T1D donors with ketoacidosis vs. without ketoacidosis (right). The genes that are shown were selected as the union of the top5 DEG for each subpopulation. Color indicates the avg. log2FC from using cell-level analysis using Wilcoxon test (default method in the Seurat package). Stars indicate statistically significant differences after Bonferroni adjustment for multiple comparisons. **(D)** Heatmaps showing the overlap of the top 100 upregulated (red color) or top 100 downregulated (blue color) genes between the subpopulations of CD4+ T cells. The top 100 genes were the 100 genes with the highest or lowest log2 FC obtained from the following contrasts: T1D donors without partial remission vs. with partial remission (left), and T1D donors with ketoacidosis vs. without ketoacidosis (right) using cell-level analysis using Wilcoxon test (default method in the Seurat package).

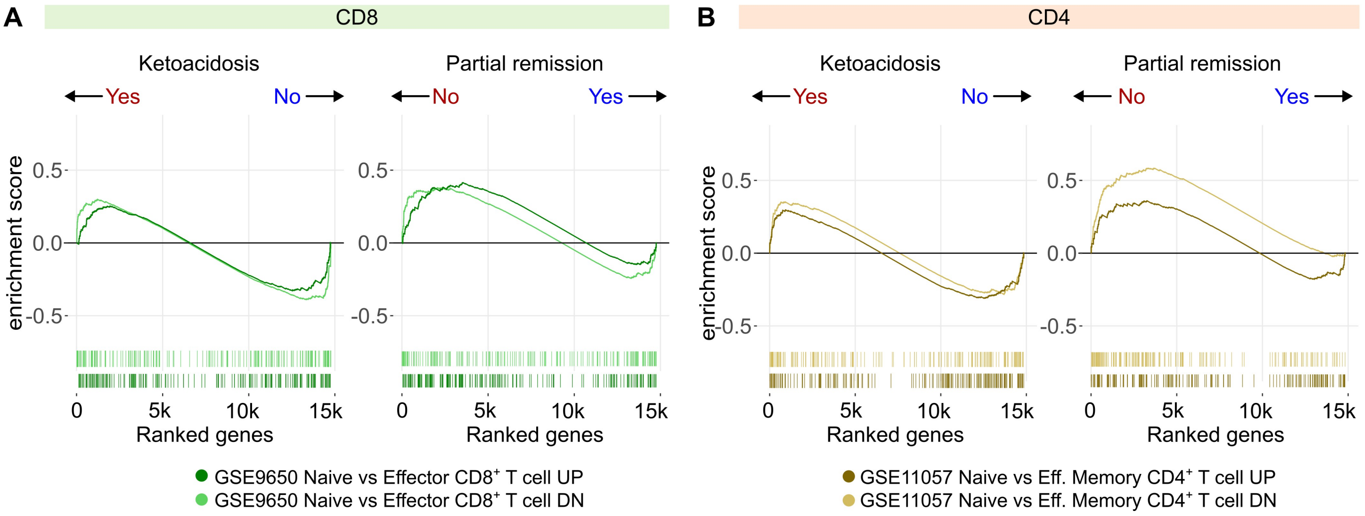

Supplementary Figure 13. GSEA analysis in ketoacidosis and partial remission. (A-B) Gene set enrichment analysis (GSEA) illustrating the enrichment of gene signatures previously associated with naïve versus effector T cell states in previously published studies (GSE9650, GSE11057) in our cohort. Each plot shows curves for two gene sets: genes upregulated in naïve versus effector cells (dark curve, "UP" gene set) and genes downregulated in naive versus effector cells (light curve, "DN" gene set) from the original studies. Positive normalized enrichment scores indicate stronger enrichment of the naïve-up gene set and depletion of the effector-up gene set in CD8+ T cells (A) or CD4+ T cells (B) from T1D donors without partial remission vs. with partial remission (left), and T1D donors with ketoacidosis vs. without ketoacidosis (right).

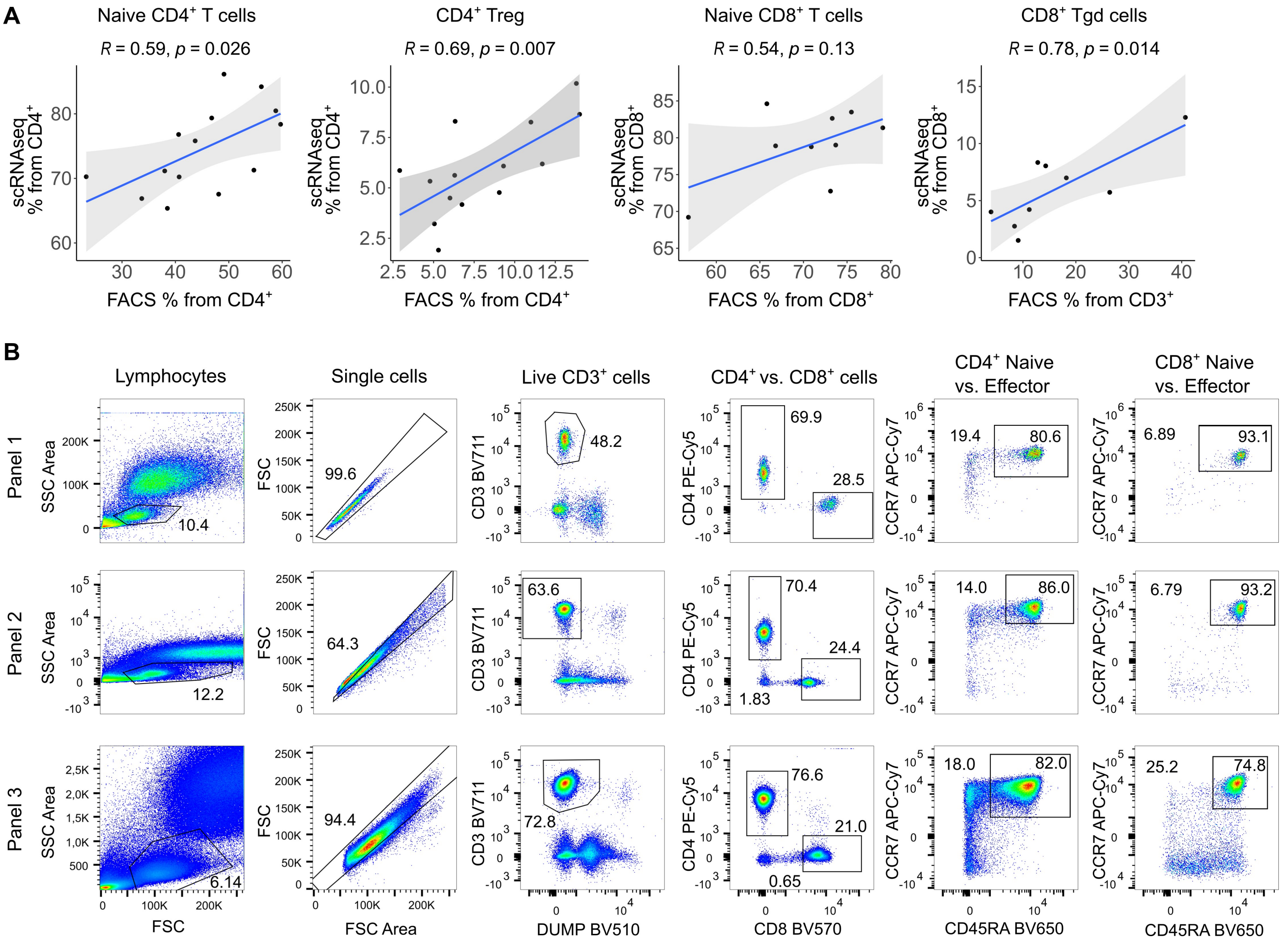

Supplementary Figure 14. Flow cytometry validation of findings from scRNAseq. (A) Correlation of the percentages of Naïve CD4+ T cells, CD4+ Treg cells, Naïve CD8+ T cells and CD8+ Tgd cells measured by scRNAseq and by flow cytometry. Each dot represents the same sample that was processed by scRNAseq (initial experiment only) and by flow cytometry. P-value was calculated using Pearson's test. Line = regression fit; shaded area = 95% CI. **(B)** Representative gating of CD4+ and CD8+ Naïve and Effector cells in flow cytometry samples from the HPAP database. In the three rows, we present the same populations gated in three different panels obtained from the original source: Panel 1 – CD4 phenotyping panel, Panel 2 – CD8 phenotyping panel and Panel 3 – CD8 phenotyping panel focused on antigen-specific cells. Samples are representatives of n = 41 sample in Panel 1, n = 19 samples in Panel 2, n = 26 samples in Panel 3.

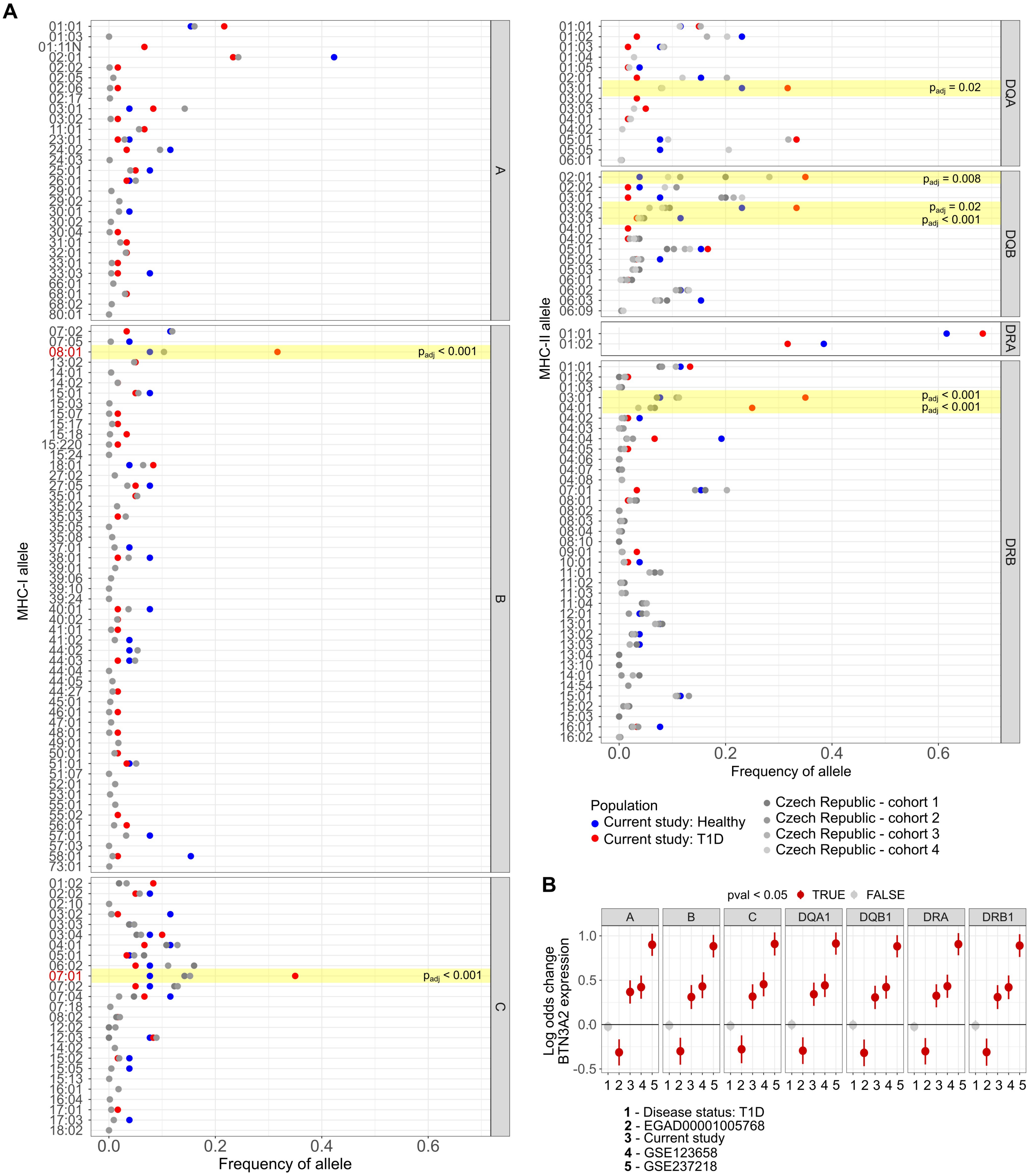

Supplementary Figure 15. Analysis of HLA alleles in children with T1D. (A) Analysis of the frequencies of HLA class I (left) and HLA class II (right) genotypes of healthy donors in the Czech Republic and participants of the current study. HLA genotypes of T1D and healthy donors were inferred from scRNAseq data using the tool ArcasHLA [5]. The frequencies of HLA alleles in the global population of the Czech Republic was obtained from The Allele Frequency Net Database. P-value was calculated using binomial test and adjusted for multiple comparison using the Bonferroni method. For testing, the frequency of the allele in global Czech population was calculated as the average of frequencies in the four Czech Republic reference datasets. **(B)** Normalized expression of BTN3A2 was compared among five studies with T1D and healthy donors. HLA genotype of donors was obtained from the original metadata or inferred from the raw RNAseq data using ArcasHLA. The effect of study, diabetes status, and particular alleles in all MHC-I and MHC-II loci was calculated using generalized linear model with gaussian distribution. Statistically significant predictor variables in the model are marked in red color. The analysis was performed for each locus separately. Shown is the effect of disease status and each of the listed studies on BTN3A2 expression. Dots represent the log odds change, whiskers represent 95% CIs.

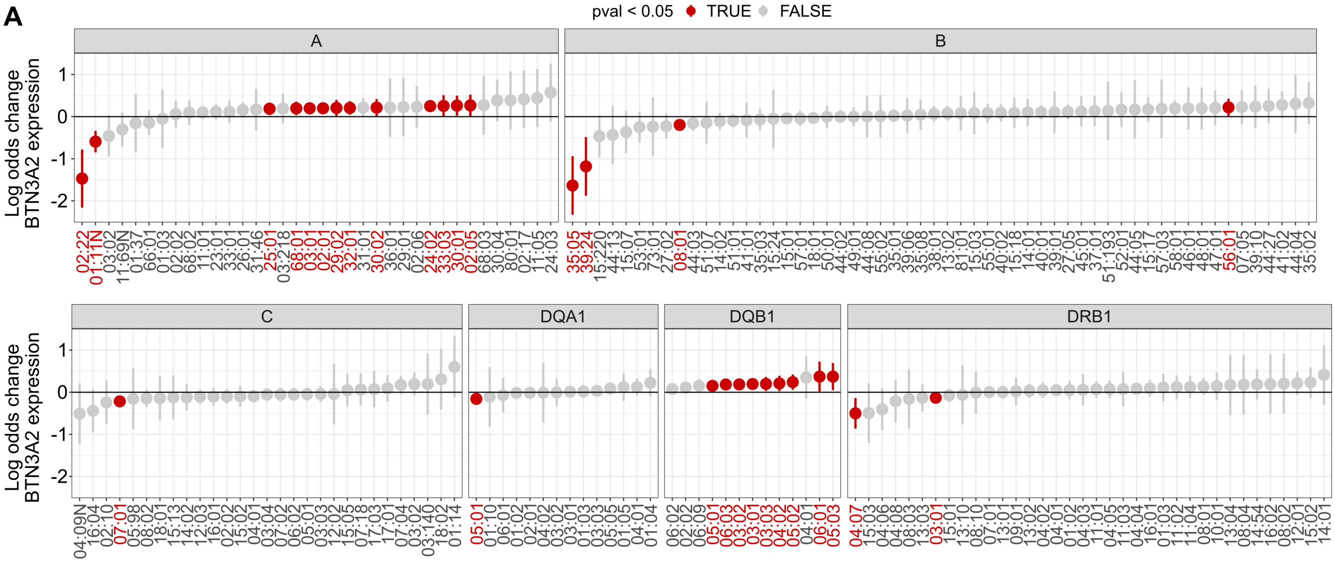

Supplementary Figure 16. BTN3A2 expression is linked to specific HLA alleles. (A) Normalized expression of BTN3A2 was compared among five studies with T1D and healthy donors. HLA genotype of donors was obtained from the original metadata or inferred from the raw RNAseq data using ArcasHLA. The effect of study, diabetes status, and particular alleles in all MHC-I and MHC-II loci was calculated using generalized linear model with gaussian distribution. Statistically significant predictor variables in the model are marked in red color. The analysis was performed for each locus separately. The effect of the MHC-I and MHC-II alleles on BTN3A2 expression is shown. Dots represent the log odds change, whiskers represent 95% CIs.

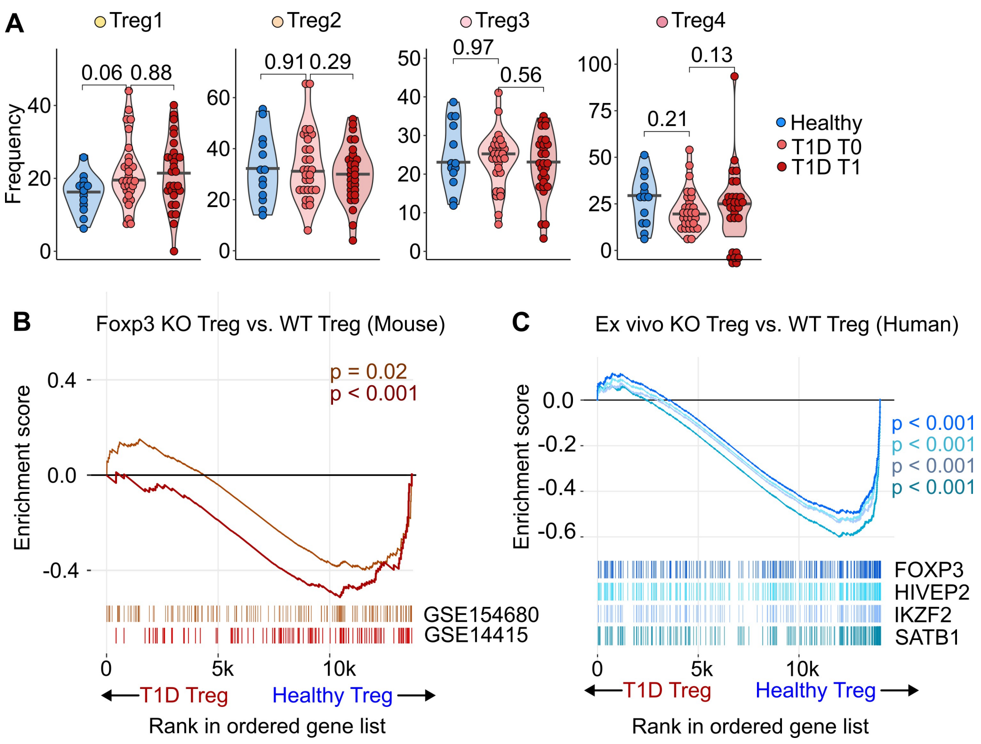

Supplementary Figure 17. Analysis of Treg cells in T1D. (A) Quantification of the cluster composition for clusters shown in Fig 3A. Violin plots show the percentage of cells in each subcluster from total Treg cells in healthy and T1D donors at T0 and T1. Bar at median. P-value was calculated using two-tailed Mann-Whitney test between healthy and T1D T0 donors, or two-tailed paired Mann-Whitney test between T1D donors at T0 and T1. **(B)** Gene set enrichment analysis (GSEA) showing the enrichment of genes identified as downregulated in Foxp3 KO mice vs. control wild-type mice [6,7] in children with T1D vs. healthy donors from our study. The ranked gene list represents the contrast between Tregs of patients with T1D vs. healthy donors. **(C)** GSEA showing the enrichment of genes identified as downregulated in human Treg cells with CRISPR-mediated KO of FOXP3, HIVEP2, IKZF2, and SATB1 vs. normal human Treg cells [8] in children with T1D vs. healthy donors from our study. The ranked gene list represents the contrast between Tregs of children with T1D vs. healthy donors.

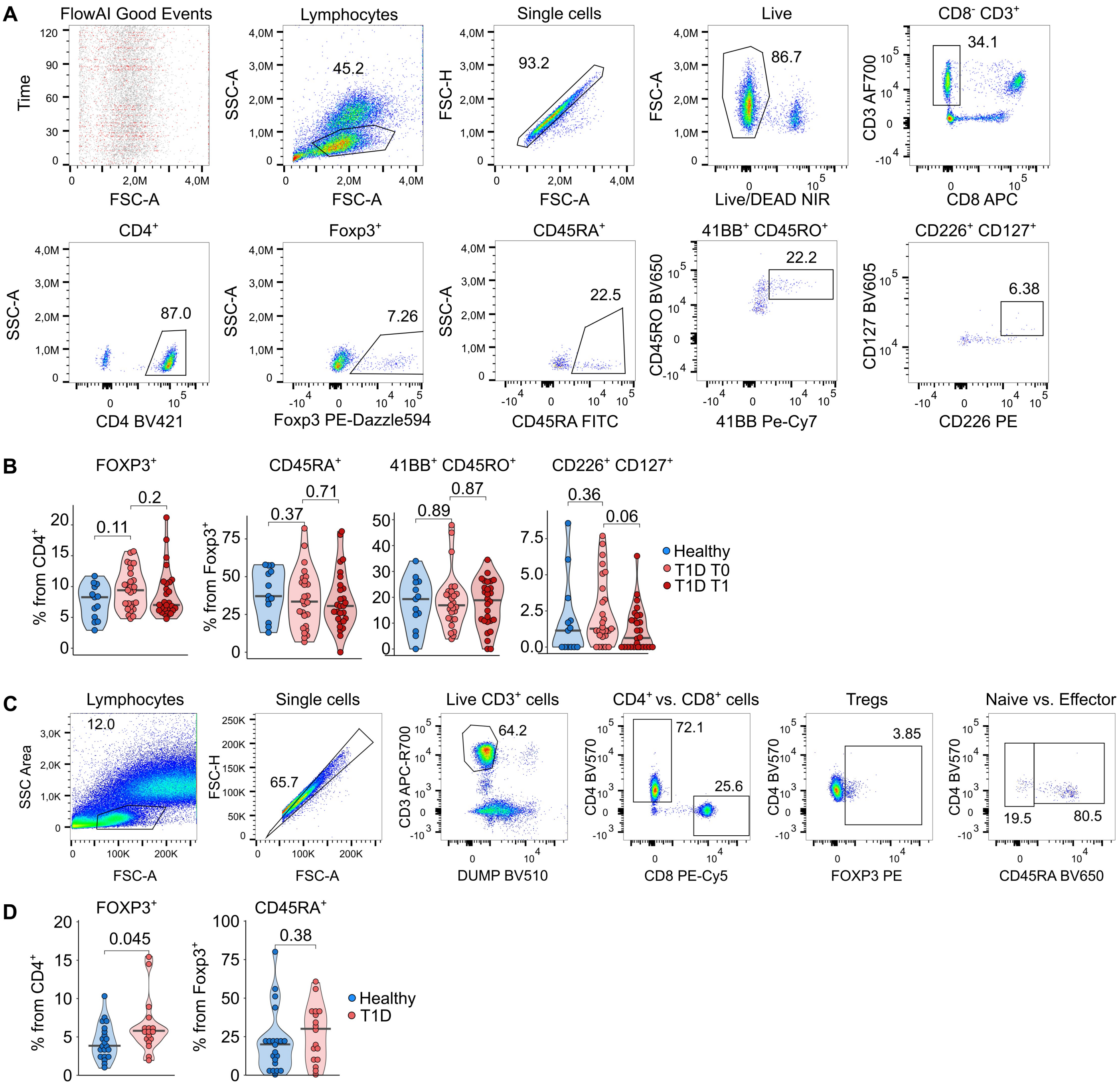

Supplementary Figure 18. Flow cytometry analysis of Treg cells in T1D. (A-B) Flow cytometry analysis of the Treg cells and Treg subpopulations in the current study. n = 13 healthy donors, n = 27 T1D T0 donors, n = 28 T1D T1 donors. **(A)** Representative flow cytometry gating of the Treg cells and Treg subpopulations in the current study shown in B). **(B)** Quantification of the Treg cells and Treg subpopulations in donors with T1D and healthy donors measured by flow cytometry. The following populations are quantified: Foxp3+ from CD4+ T cells, CD45RA+ from Foxp3+ CD4+ T cells, 4-1BB+ CD45RO+ from Foxp3+ CD4+ T cells, CD45RA+ CD226+ CD127+ from Foxp3+ CD4+ T cells. Representative gating is shown in (A). P-value was calculated using two-tailed Mann-Whitney test between healthy and T1D T0 donors, or two-tailed paired Mann-Whitney test between T1D donors at T0 and T1. **(C-D)** Flow cytometry analysis of the Treg cells and Treg subpopulations in the HPAP dataset. n = 21 non-diabetic donors, n = 17 T1D donors. **(C)** Representative flow cytometry gating of the Treg cells and Treg subpopulations in the HPAP dataset. **(D)** Quantification of the Foxp3+ from CD4+ T cells and CD45RA+ Foxp3+ CD4+ T cells in donors with T1D and healthy donors from the HPAP database. Representative gating is shown in (C). P-value was calculated using two-tailed Mann-Whitney test. Bar at median.

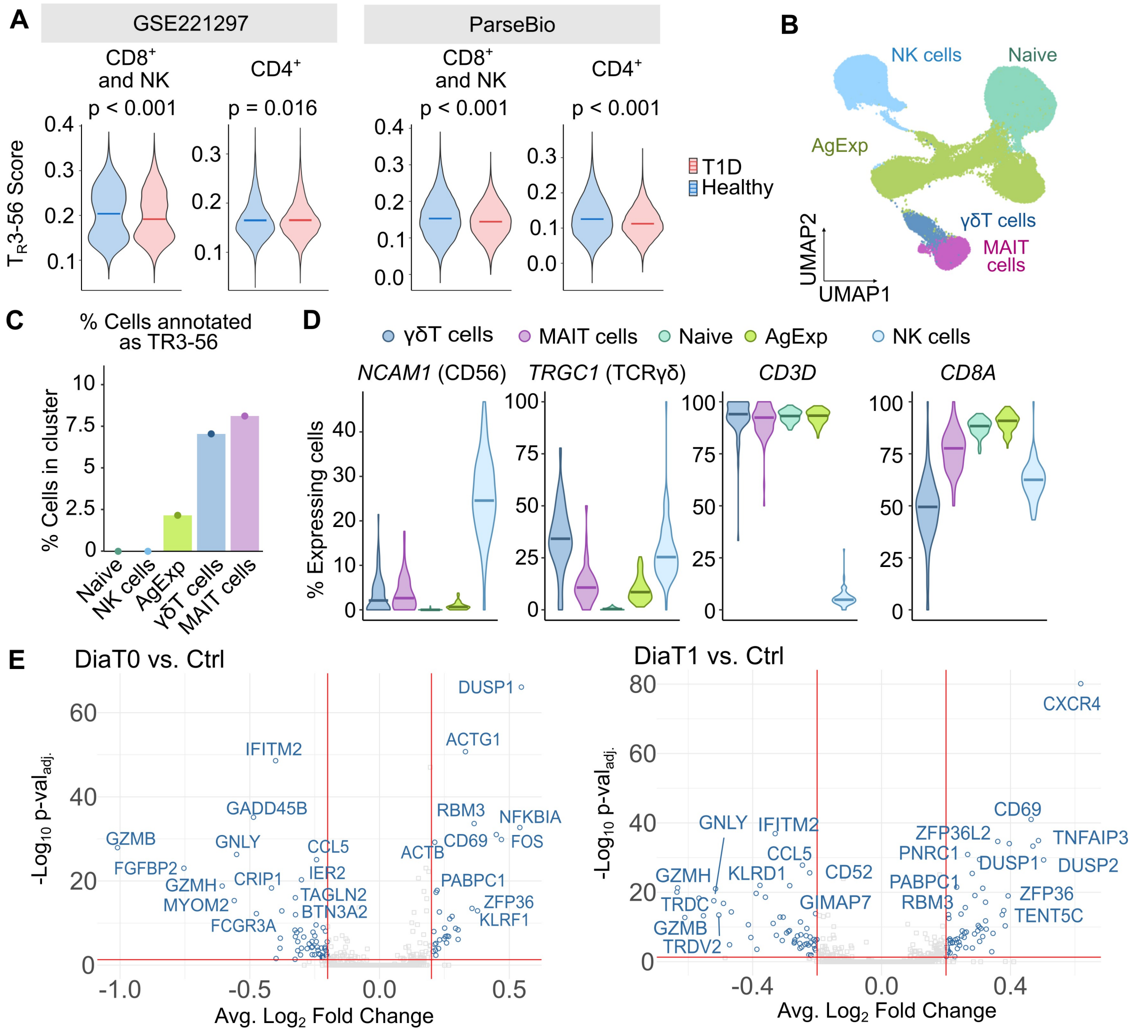

Supplementary Figure 19. Analysis of unconventional CD8+ T cells. (A) Cells from the datasets GSE221297 and ParseBio were annotated with a previously published dataset of FACS-sorted TR3-56 (CD3+ CD56-) cells, NK cells, CD3+ CD56-, and CD8+ cells (GSE106082 [9] using the SingleR package. The violin plots show quantification of the annotation scores of TR3-56 cells (i.e., similarity of the gene expression of a particular cell to that of TR3-56 cells) in CD8+ and NK or CD4+ T cells in healthy and T1D donors. GSE221297 CD4+: n = 35,050 cells from 8 donors, GSE221297 CD8+ and NK: n = 38,243 cells from 8 donors, ParseBio CD4+: n = 63,219 cells from 24 donors, ParseBio CD8+ and NK: n = 49,844 cells from 24 donors. P-value was calculated using two-tailed Mann-Whitney test. Bar at median. **(B)** UMAP projection of CD8+ T cells showing groups of cells used for the analysis of unconventional CD8+ subsets. Louvain clusters were merged based on functional relevance. n = 95,229 cells from 43 donors. **(C)** Violin plots showing the percentage of cells from the clusters indicated in (B) that have non-zero expression of the selected gene within the specified cluster. Data is based on expression profiles from 87 samples. Bar at median. **(D)** Percentage of cells in each cluster indicated in (B) annotated as TR3-56 cells. **(E)** Volcano plot showing differentially expressed genes in Unconventional CD8+ T cells from patients with T1D sampled at their diagnosis compared to healthy donors (left) or from one-year follow-up samples of the same patients compared to healthy donors (right). The average log2 fold changes were calculated using the FindMarkers function from the Seurat package (Wilcoxon test). Multiple-hypothesis testing is controlled using the Bonferroni correction.

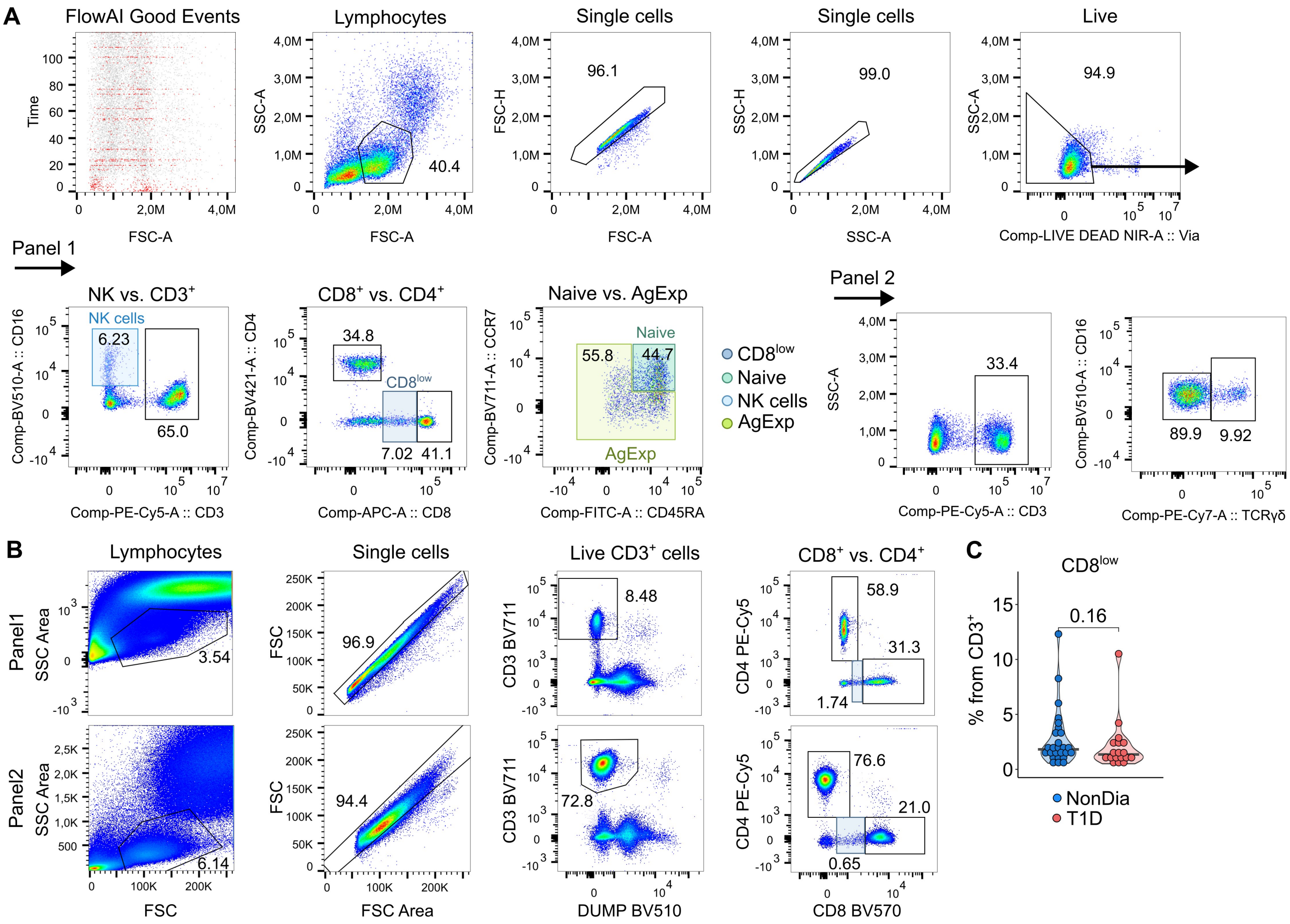

Supplementary Figure 20. Flow cytometry analysis of CD8low T cells and Tgd cells. (A) Representative flow cytometry gating of the populations of cells shown in Fig. 6J-K. **(B)** Flow cytometry analysis of CD8low T cells from the PBMC of deceased donors with T1D compared to healthy donors from the HPAP database. Representative flow cytometry gating of CD8low T cells in the HPAP dataset. **(C)** Quantification of CD8low T cells in T1D patients and healthy donors. P-value was calculated using two-tailed Mann-Whitney test.

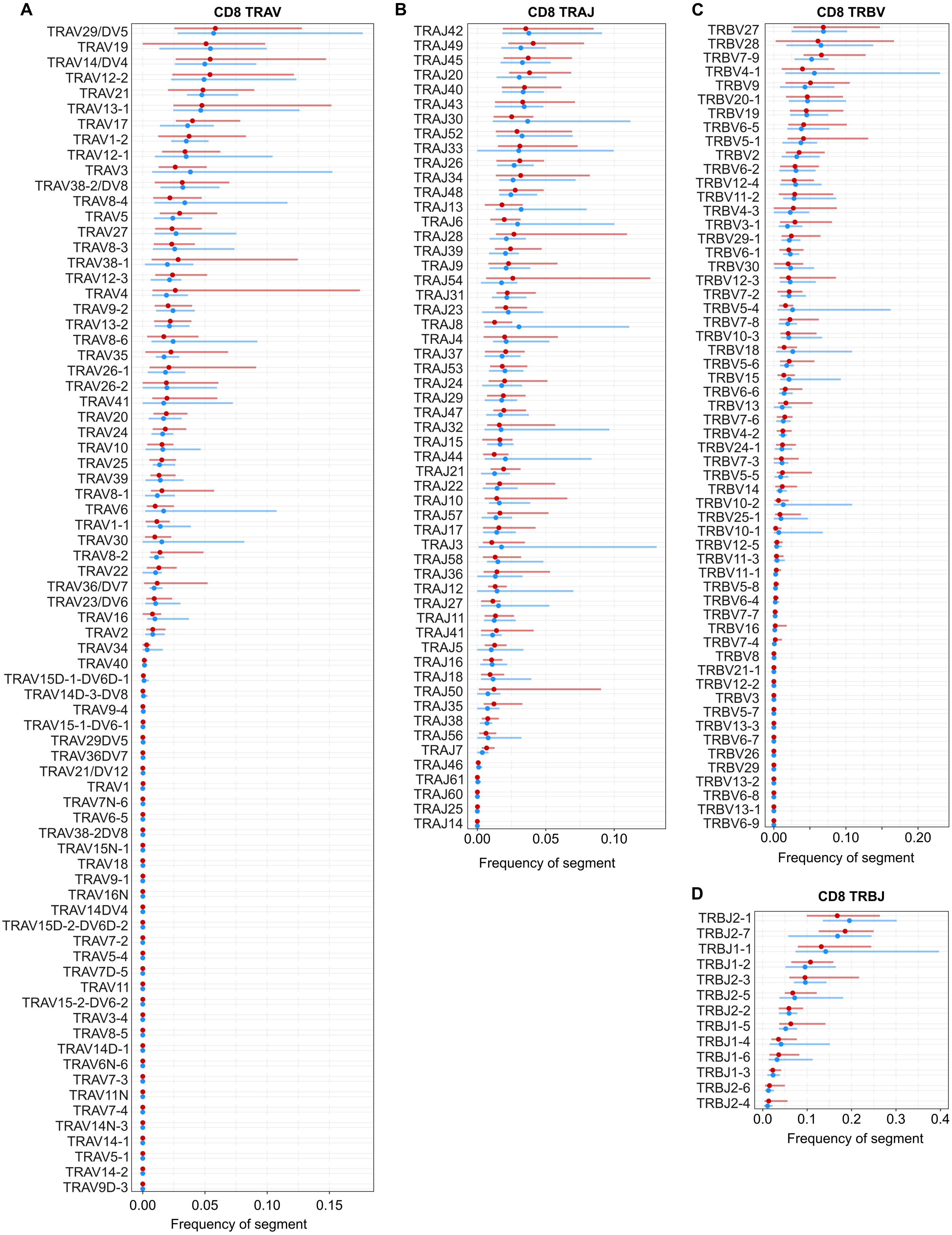

Supplementary Figure 21. Analysis of TCR repertoires in CD8+ T cells. Analysis of gene segment usage of TRAV (A), TRAJ (B), TRBV (C) and TRBJ (D) genes in conventional CD8+ T cells from healthy (blue) and T1D (red) donors. TCR repertoires were profiled using 10x Immune Profiling with Feature Barcoding Technology. Dot represents mean, whiskers range from min to max.

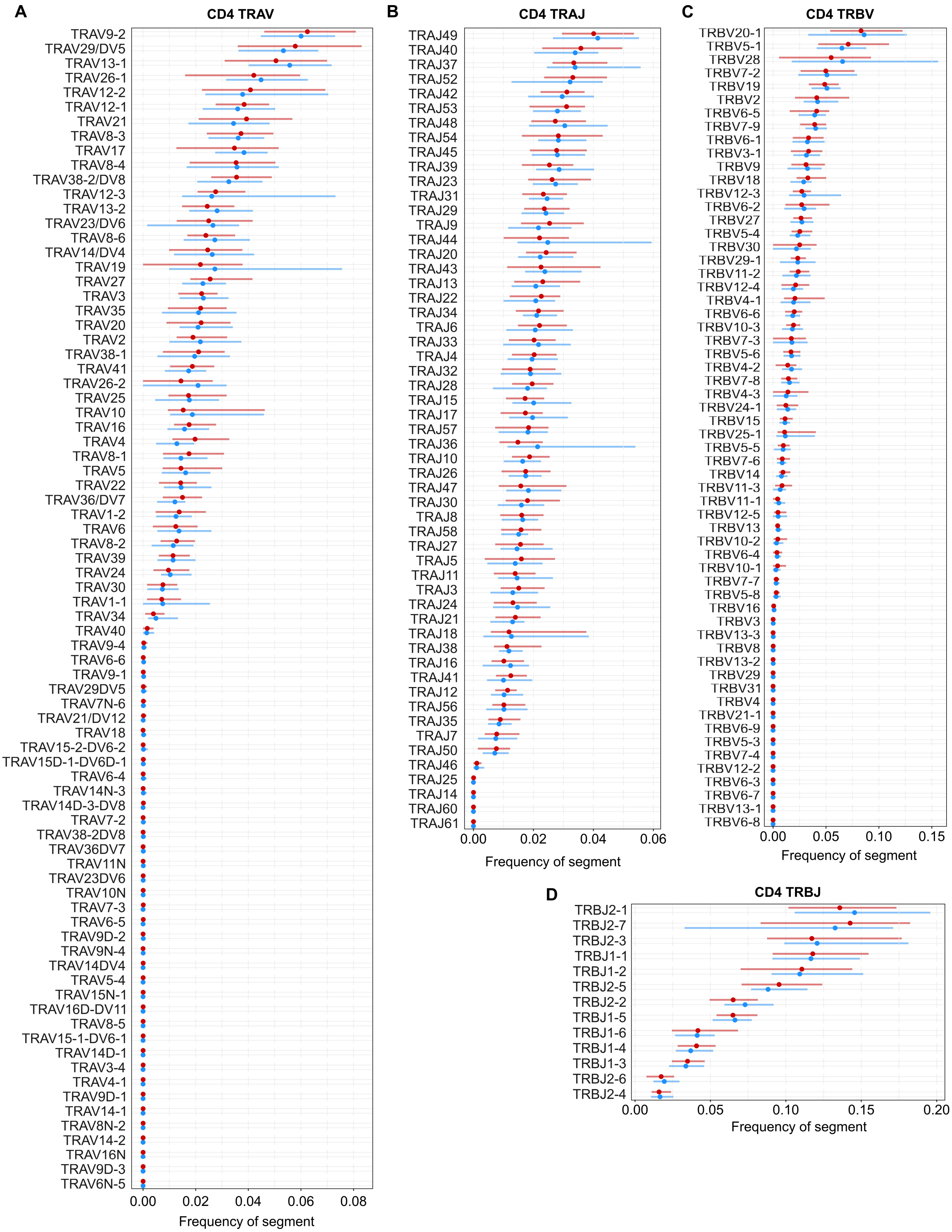

Supplementary Figure 22. Analysis of TCR repertoires in CD4+ T cells. Analysis of gene the segment usage of TRAV (A), TRAJ (B), TRBV (C) and TRBJ (D) genes in conventional CD4+ T cells from healthy (blue) and T1D (red) donors. TCR repertoires were profiled using 10x Immune Profiling with Feature Barcoding Technology. Dot represents mean, whiskers range from min to max.

References to Supplementary Information

1 Kitaura, K., Shini, T., Matsutani, T. & Suzuki, R. A new high-throughput sequencing method for determining diversity and similarity of T cell receptor (TCR) α and β repertoires and identifying potential new invariant TCR α chains. *BMC immunology* **17**, 38, doi:10.1186/s12865-016-0177-5 (2016).

2 Müller-Dott, S. *et al.* Expanding the coverage of regulons from high-confidence prior knowledge for accurate estimation of transcription factor activities. *Nucleic Acids Research* **51**, 10934-10949, doi:10.1093/nar/gkad841 (2023).

3 Michalik, J., Niederlova, V. & Stepanek, O. IDEIS: a tool to identify PTPRC/CD45 isoforms from single-cell transcriptomic data. *Frontiers in immunology* **15**, doi:10.3389/fimmu.2024.1446931 (2024).

4 Giles, J. R. *et al.* Human epigenetic and transcriptional T cell differentiation atlas for identifying functional T cell-specific enhancers. *Immunity* **55**, 557-574.e557, doi:10.1016/j.immuni.2022.02.004 (2022).

5 Orenbuch, R. *et al.* arcasHLA: high-resolution HLA typing from RNAseq. *Bioinformatics* **36**, 33-40, doi:10.1093/bioinformatics/btz474 (2020).

6 van der Veeken, J. *et al.* The Transcription Factor Foxp3 Shapes Regulatory T Cell Identity by Tuning the Activity of trans-Acting Intermediaries. *Immunity* **53**, 971-984.e975, doi:10.1016/j.immuni.2020.10.010 (2020).

7 Haribhai, D. *et al.* A central role for induced regulatory T cells in tolerance induction in experimental colitis. *J Immunol* **182**, 3461-3468, doi:10.4049/jimmunol.0802535 (2009).

8 Schumann, K. *et al.* Functional CRISPR dissection of gene networks controlling human regulatory T cell identity. *Nat Immunol* **21**, 1456-1466, doi:10.1038/s41590-020-0784-4 (2020).

9 Terrazzano, G. *et al.* T1D progression is associated with loss of CD3(+)CD56(+) regulatory T cells that control CD8(+) T cell effector functions. *Nat Metab* **2**, 142-152, doi:10.1038/s42255-020-0173-1 (2020).

Variable	Group	Time	n	NA count	Mean	SD	T1D T0 vs Healthy Wilcox. p-val
**Age (years)**	Healthy	T0	13	0	9.59	5.82	p = 1
	T1D	T0	30	0	9.64	5.04	
	T1D	T1	29	1	10.87	4.94	
**BMI (kg/m2)**	Healthy	T0	11	2	18.71	3.04	p = 0.13
	T1D	T0	30	0	17.31	4.18	
	T1D	T1	29	1	19.74	3.93	
**Fasting C-peptide (pmol/L)**	Healthy	T0	11	2	642.91	296.16	p = 5.09e-06
	T1D	T0	30	0	176.59	181.54	
	T1D	T1	28	2	221.89	227.88	
**Hba1c (mmol/mol)**	Healthy	T0	11	2	33.55	3.14	p = 1.45e-06
	T1D	T0	29	1	109.31	17.72	
	T1D	T1	28	2	44.64	5.88	

Supplementary Table 1. Characteristics of the cohort – numeric variables. P-values were calculated using the two-tailed Mann–Whitney test.

Variable	Group	Subgroup	n	Group %
**Sex**	Healthy	F	9	69.23
		M	4	30.77
	T1D	F	10	33.33
		M	20	66.67
**HLA-DQ2**	Healthy	DQ2	1	7.69
		Other	12	92.31
	T1D	DQ2	16	53.33
		Other	14	46.67
**HLA-DQ8**	Healthy	DQ8	4	30.77
		Other	9	69.23
	T1D	DQ8	17	56.67
		Other	13	43.33
**HLA-DQ2 and DQ8**	Healthy	DQ2_8	0	0.00
		Other	8	100.00
	T1D	DQ2_8	7	23.33
		Other	23	76.67

Supplementary Table 2. Characteristics of the cohort – categoric variables.

Batch	Sort strategy	Enrichment	Count of donors						Count of 10x wells
			CD8+ T cells			CD4+ T cells			
Initial experiment			T1D T0	T1D T1	HD	T1D T0	T1D T1	HD	
Batch 1	CD4+ or CD8+	No enrichment	6						1
Batch 2	CD4+ or CD8+	No enrichment	6		4	12		4	3
Final experiment									
Batch 3	CD4+ or CD8+, CD45RA, CCR7	Naïve*:AgExp 1:5	8	8	3	8	8	3	4
Batch 4	CD4+ or CD8+, CD45RA, CCR7	Naïve*:AgExp 1:5	8	8	4	8	8	4	4
Batch 5	CD4+ or CD8+, CD45RA, CCR7	Naïve*:AgExp 1:5	8	7	2	8	7	2	4
Batch 6	CD4+ or CD8+, CD45RA, CCR7	Naïve*:AgExp 1:5	6	6	4	6	6	4	4

* Naïve cells sorted as CD45RA+CCR7+

Supplementary Table 3. List of performed experiments, enrichment strategies and sample counts.

Cell type	Batch	Well	Reads per cell GEX (mean)	Reads per cell VDJ (mean)	Count of detected genes per cell averaged by sample (mean ± SD)	Count of UMIs per cell averaged by sample (mean ± SD)	Count of cells per sample (mean ± SD)
CD8	Batch1	Well1	58620	4005	1157 ± 93	2111 ± 253	897 ± 53
CD8	Batch2	Well1	19219	2450	1053 ± 46	1853 ± 116	1067 ± 53
CD8	Batch3	Well1	53849	3369	1764 ± 99	3355 ± 295	758 ± 96
		Well2	50016	4716	1749 ± 100	3353 ± 326	845 ± 26
CD8	Batch4	Well1	21572	3803	1575 ± 71	2892 ± 191	1373 ± 47
		Well2	35265	3045	1701 ± 146	3355 ± 703	1381 ± 227
CD8	Batch5	Well1	38958	3747	1476 ± 89	2683 ± 291	1185 ± 52
		Well2	40454	5174	1594 ± 76	2894 ± 207	1233 ± 380
CD8	Batch6	Well1	29253	2969	1274 ± 67	2185 ± 151	1217 ± 68
		Well2	69146	4699	1656 ± 96	3031 ± 251	850 ± 56
CD4	Batch2	Well2	60630	4056	985 ± 59	1678 ± 91	775 ± 27
		Well3	28427	3422	948 ± 32	1611 ± 86	756 ± 227
CD4	Batch3	Well3	63515	3555	1698 ± 113	3203 ± 304	725 ± 73
		Well4	82769	4746	1750 ± 92	3377 ± 343	756 ± 37
CD4	Batch4	Well3	23639	3023	1616 ± 55	3018 ± 147	1189 ± 113
		Well4	27138	3061	1518 ± 107	2822 ± 254	1258 ± 144
CD4	Batch5	Well3	44719	4383	1423 ± 64	2564 ± 177	1024 ± 60
		Well4	30408	3311	1466 ± 77	2658 ± 203	1065 ± 477
CD4	Batch6	Well3	50195	3171	1156 ± 35	1946 ± 62	868 ± 111
		Well4	87115	3535	1716 ± 82	3183 ± 229	618 ± 43
**Mean ± SD**			**45745 ± 19431**	**3712 ± 709**	**1464 ± 266**	**2689 ± 578**	**992 ± 230**

Supplementary Table 4. List of sequencing runs and per-cell metrics in different batches.

	Study	Reposited data	Participants	Cell type	Method
Bulk transcriptomics	Newman et al., 2023	GSE237218	CD4+/CD25+ T cells: 49 T1D cases, 35 controls<br>CD4+/CD25− T cells: 53 T1D cases, 52 controls<br>Memory CD4+ T cells: 19 T1D cases, 27 controls	Sorted T cell subsets	Bulk RNAseq
	Valentim et al., 2018 *	GSE123658	39 T1D cases, 43 healthy donors	Whole blood, hemoglobin depleted	Bulk RNAseq
	Kallionpää et al., 2019	EGAD00001005767	7 T1D cases, 8 controls	Sorted T cell subsets	Bulk RNAseq
	Jailwala et al., 2009	GSE10586	12 T1D cases, 15 healthy donors	Sorted T cell subsets	Microarray
Single-cell transcriptomics	HPAP - scRNAseq *	https://hpap.pmacs.upenn.edu/	4 T1D cases, 8 controls	Splenocytes	scRNAseq
	Kallionpää et al., 2019	EGAD00001005768	4 T1D cases, 4 controls	PBMC	scRNAseq
	Honardoost et al., 2024	syn53641849	46 T1D cases, 31 controls	PBMC	scRNAseq
	ParseBio *	https://resources.parsebiosciences.com/	12 T1D, 12 healthy donors	PBMC	scRNAseq
	Zhong et al., 2024	GSE221297	5 new onset, 3 healthy controls	PBMC	scRNAseq
TCR profiling	HPAP - TCRseq *	https://hpap.pmacs.upenn.edu/	16 T1D cases, 38 controls	Splenocytes	TCRseq
FACS	HPAP - FACS *	https://hpap.pmacs.upenn.edu/	23 T1D cases, 32 controls	PBMC	FACS

* Unpublished dataset

Supplementary Table 5. List of transcriptomics datasets from public resources and previously published studies [9-13] used for validation of the findings.

Name	Colour	Cat. Num.	Producer	Clone	LOT	Dilution
**Antibodies used for flow cytometry analysis**						
CD4	BV 421	344632	BioLegend	SK3	B202013	200x
CD45R/B220	BV 510	103248	BioLegend	RA3-6B2	B365977	200x
CD127	BV605	351333	BioLegend	A019D5	B384035	300x
CD45RO	BV 650	304232	BioLegend	UCHL1	B401941	200x
Ki-67	BV750	350535	BioLegend	Ki-67	B406901	1000x
CD45RA	FITC	1F-223-T100	Exbio	MEM-56	537897, 535222	200x
HLA-DR	PerCP/Cy5.5	327019	BioLegend	LN3	B392220	500x
CD226	PE	1P-926-T100	Exbio	11A8	542427	200x
CD25	PE/Fire700	356146	BioLegend	M-A251	B376207	200x
FOXP3	PE/Dazzle 594	320125	BioLegend	206D	B400205	500x
CD137	PE-Cy7	25-1379-42	eBioscience	4B4	2450669	500x
CD8	APC	1A-207-T100	Exbio	MEM-31	530450	100x
CD3	AF 700	A7-202-T100	Exbio	MEM-57	2175105	100x
IκBα	AF 488	5743S	Cell Signaling	L35A5	12	300x
Granzyme K	PerCP/Cy5.5	370513	BioLegend	GM26E7	B389008	100x
CD184	PE	1P-146-T100	Exbio	12G5	542607	200x
EOMES	PE/CF594	567167	BD Pharmingen	X4-83	3187264	500x
Granzyme B	PE/Cy7	372213	BioLegend	QA16A02	B368221	500x
CD16	BV510	360729	BioLegend	B73.1	B406541	100x
TCR Vα24-Jα18	BV605	342929	BioLegend	6B11	B350837	100x
CD197	BV711	353227	BioLegend	G043H7	B303460	200x
CD3	PE-Cy5	561007	BD	UCHT1	2175105	100x
TCR gamma/delta	PE-Cy7	T7-912-T100	Exbio	11F2	544439	200x
CD56	APC-R700	565140	BD	NCAM16.2	2301665	200x
**Antibodies used for cell sorting**						
CD4	APC	1A-359-T100	Exbio	MEM-31	540684	100x
CD8	PE	1P-207-T025	Exbio	MEM-31	541174	100x
CD45RA	FITC	1F-223-T100	Exbio	MEM-56	537897	200x
CD197	PE-Cy7	353226	BioLegend	G043H7	B305236	200x
CD8	APC	1A-817-T100	Exbio	LT8	546128	100x
CD4	AF 700	A7-539-T100	Exbio	MEM-241	545149	100x
**Antibodies used for cell hashing**						
TotalSeq™-C0251 anti-human Hashtag 1 Antibody		394661	BioLegend	LNH-94 2M2	B431103	100x
TotalSeq™-C0252 anti-human Hashtag 2 Antibody		394663	BioLegend	LNH-94 2M2	B294923	100x
TotalSeq™-C0253 anti-human Hashtag 3 Antibody		394665	BioLegend	LNH-94 2M2	B342838	100x
TotalSeq™-C0254 anti-human Hashtag 4 Antibody		394667	BioLegend	LNH-94 2M2	B301957	100x
TotalSeq™-C0255anti-human Hashtag 5 Antibody		394669	BioLegend	LNH-94 2M2	B343533	100x
TotalSeq™-C0256 anti-human Hashtag 6 Antibody		394671	BioLegend	LNH-94 2M2	B306449	100x
TotalSeq™-C0257 anti-human Hashtag 7 Antibody		394673	BioLegend	LNH-94 2M2	B306454	100x
TotalSeq™-C0258 anti-human Hashtag 8 Antibody		394675	BioLegend	LNH-94 2M2	B306430	100x
TotalSeq™-C0259 anti-human Hashtag 9 Antibody		394677	BioLegend	LNH-94 2M2	B309896	100x
TotalSeq™-C0260 anti-human Hashtag 10 Antibody		394679	BioLegend	LNH-94 2M2	B309891	100x
**Viability and FC blocking**						
LIVE/DEAD NIR		L34976	Thermo Fisher Scientific		3079936	500x
Hoechst 33258		H3569	Thermo Fisher Scientific			1000x
Human TruStain FcX		422302	BioLegend		B382097	500x

Supplementary Table 6. List of used flow cytometry antibodies and viability dyes.

Panel 1 - CD4 global panel								
Included	Donor ID	RRID	Sex	BMI	Age	hba1c	Diagnosis	Exclusion reason
✓	**HPAP-008**	RRID:SAMN19776440	F	31,9	24	5,2	Control	
✓	**HPAP-017**	RRID:SAMN19776448	M	23,7	30	5,5	Control	
✓	**HPAP-021**	RRID:SAMN19776452	F	21,4	13		T1DM	
✓	**HPAP-022**	RRID:SAMN19776453	F	34,7	39	4,7	Control	
✓	**HPAP-023**	RRID:SAMN19776454	F	21,35	17	8,9	T1DM	
✓	**HPAP-024**	RRID:SAMN19776455	M	24,3	18	5,5	Control	
✓	**HPAP-025**	RRID:SAMN19776456	M	33,4	30	8,9	T1DM	
✓	**HPAP-026**	RRID:SAMN19776457	M	20,8	24	4,9	Control	
✓	**HPAP-027**	RRID:SAMN19776458	F	32,71	31	4,4	Control	
✓	**HPAP-029**	RRID:SAMN19776460	M	28,6	23	5,3	Control	
✓	**HPAP-030**	RRID:SAMN19776461	M	21,3	18	12,4	T1DM	
✓	**HPAP-031**	RRID:SAMN19776462	M	22,64	23	6,8	T1DM	
✓	**HPAP-032**	RRID:SAMN19776463	F	16,3	10	9	T1DM	
✓	**HPAP-034**	RRID:SAMN19776465	M	18,6	13	5,2	Control	
✓	**HPAP-035**	RRID:SAMN19776466	M	26,91	35	5,2	Control	
✓	**HPAP-036**	RRID:SAMN19776467	F	16	23	5,2	Control	
✓	**HPAP-037**	RRID:SAMN19776468	F	21,9	35	5,3	Control	
✓	**HPAP-041**	RRID:SAMN19776472	F	20,93	24	7,6	T1DM	
✓	**HPAP-046**	RRID:SAMN19776477	M	20,96	19	5,7	Control	
✓	**HPAP-047**	RRID:SAMN19776478	M	16,82	8		Control	
✓	**HPAP-049**	RRID:SAMN19776480	M	37,2	29	5,4	Control	
✓	**HPAP-050**	RRID:SAMN19776481	F	28,99	21	5,1	Control	
✓	**HPAP-060**	RRID:SAMN19842589	M	26,2	30	4,9	Control	
✓	**HPAP-064**	RRID:SAMN19842593	M	16,98	24	13	T1DM	
✓	**HPAP-068**	RRID:SAMN19842597	F	30,85	20	13,9	T1DM	
✓	**HPAP-071**	RRID:SAMN19842600	F	15,42	12	9,8	T1DM	
✓	**HPAP-072**	RRID:SAMN19842601	M	23,1	19	5,6	Control	
✓	**HPAP-073**	RRID:SAMN19842602	M	25,92	24	8,2	T1DM	
✓	**HPAP-084**	RRID:SAMN19842613	F	18,5	12	13,3	T1DM	
✓	**HPAP-087**	RRID:SAMN19842616	F	19,3	15	10,4	T1DM	
✓	**HPAP-092**	RRID:SAMN19842621	M	25,59	21	5,6	Control	
✓	**HPAP-094**	RRID:SAMN22562805	F	37,84	38	5	T1DM	
✓	**HPAP-095**	RRID:SAMN22562806	F	34,32	23	4,9	Control	
✓	**HPAP-098**	RRID:SAMN22562809	F	34,9	30	11	T1DM	
✓	**HPAP-099**	RRID:SAMN22562810	F	24,7	28	5	Control	
✓	**HPAP-102**	RRID:SAMN22562813	M	20,65	18	6,7	T1DM	
✓	**HPAP-110**	RRID:SAMN26024925	M	36,29	31	5,4	Control	
✓	**HPAP-113**	RRID:SAMN28088751	M	15,92	9	11,1	T1DM	
✓	**HPAP-114**	RRID:SAMN28088752	F	25,66	21	5,3	Control	
✓	**HPAP-122**	RRID:SAMN29419888	F	30,05	21	5,5	Control	
✓	**HPAP-130**	RRID:SAMN31536831	F	32,65	32	8	T1DM	
×	**HPAP-039**	RRID:SAMN19776470	F	16,3	5	6,8	Control	missing age-matched child with T1D
×	**HPAP-042**	RRID:SAMN19776473	M	17,9	1	5,6	Control	missing age-matched child with T1D
×	**HPAP-104**	RRID:SAMN22562815	M	20,63	4	4,9	Control	missing age-matched child with T1D
×	**HPAP-131**	RRID:SAMN31536832	M	22,5	23	5,1	Control	no staining for CD8
×	**HPAP-132**	RRID:SAMN32543540	F	15,71	5		Control	no staining for CD8
×	**HPAP-139**	RRID:SAMN33611450	M	26,52	22	5,2	Control	no staining for CD8
×	**HPAP-146**	RRID:SAMN35774092	M	28,31	27	5,5	Control	no staining for CD8

Panel 2 - CD8, global panel								
Included	Donor ID	RRID	Sex	BMI	Age	hba1c	Diagnosis	Exclusion reason
✓	**HPAP-008**	RRID:SAMN19776440	F	31,9	24	5,2	Control	
✓	**HPAP-017**	RRID:SAMN19776448	M	23,7	30	5,5	Control	
✓	**HPAP-035**	RRID:SAMN19776466	M	26,91	35	5,2	Control	
✓	**HPAP-046**	RRID:SAMN19776477	M	20,96	19	5,7	Control	
✓	**HPAP-047**	RRID:SAMN19776478	M	16,82	8		Control	
✓	**HPAP-049**	RRID:SAMN19776480	M	37,2	29	5,4	Control	
✓	**HPAP-050**	RRID:SAMN19776481	F	28,99	21	5,1	Control	
✓	**HPAP-060**	RRID:SAMN19842589	M	26,2	30	4,9	Control	
✓	**HPAP-068**	RRID:SAMN19842597	F	30,85	20	13,9	T1DM	

√	**HPAP-089**	RRID:SAMN19842618	F	21,72	27	10,4	T1DM	
√	**HPAP-092**	RRID:SAMN19842621	M	25,59	21	5,6	Control	
√	**HPAP-102**	RRID:SAMN22562813	M	20,65	18	6,7	T1DM	
√	**HPAP-107**	RRID:SAMN25600003	M	23,59	15	5,3	Control	
√	**HPAP-110**	RRID:SAMN26024925	M	36,29	31	5,4	Control	
√	**HPAP-136**	RRID:SAMN32643784	M	27,24	29	5,4	Control	
√	**HPAP-137**	RRID:SAMN32643785	M	18,85	23	16,1	T1DM	
√	**HPAP-141**	RRID:SAMN33611451	M	31,4	29	5,7	T1DM	
√	**HPAP-146**	RRID:SAMN35774092	M	28,31	27	5,5	Control	
√	**HPAP-148**	RRID:SAMN35774094	M	14,9	7	5,3	Control	
×	**HPAP-039**	RRID:SAMN19776470	F	16,3	5	6,8	Control	missing age-matched child with T1D
×	**HPAP-095**	RRID:SAMN22562806	F	34,32	23	4,9	Control	data disturbance in CD4+CD8low population
×	**HPAP-099**	RRID:SAMN22562810	F	24,7	28	5	Control	data disturbance in CD4+CD8low population
×	**HPAP-130**	RRID:SAMN31536831	F	32,65	32	8	T1DM	data disturbance in CD4+CD8low population
×	**HPAP-132**	RRID:SAMN32543540	F	15,71	5		Control	missing age-matched child with T1D

**Panel 3 - CD8, antigen specific cells**								
**Included**	**Donor ID**	**RRID**	**Sex**	**BMI**	**Age**	**hba1c**	**Diagnosis**	**Exclusion reason**
√	**HPAP-003**	RRID:SAMN18741941	M	24,5	29	5,6	Control	
√	**HPAP-005**	RRID:SAMN19776438	F	24,1	14	5,4	Control	
√	**HPAP-016**	RRID:SAMN19776447	M	25,21	30	5	Control	
√	**HPAP-020**	RRID:SAMN19776451	M	13,2	14		T1DM	
√	**HPAP-023**	RRID:SAMN19776454	F	21,35	17	8,9	T1DM	
√	**HPAP-024**	RRID:SAMN19776455	M	24,3	18	5,5	Control	
√	**HPAP-025**	RRID:SAMN19776456	M	33,4	30	8,9	T1DM	
√	**HPAP-026**	RRID:SAMN19776457	M	20,8	24	4,9	Control	
√	**HPAP-027**	RRID:SAMN19776458	F	32,71	31	4,4	Control	
√	**HPAP-030**	RRID:SAMN19776461	M	21,3	18	12,4	T1DM	
√	**HPAP-031**	RRID:SAMN19776462	M	22,64	23	6,8	T1DM	
√	**HPAP-032**	RRID:SAMN19776463	F	16,3	10	9	T1DM	
√	**HPAP-034**	RRID:SAMN19776465	M	18,6	13	5,2	Control	
√	**HPAP-036**	RRID:SAMN19776467	F	16	23	5,2	Control	
√	**HPAP-037**	RRID:SAMN19776468	F	21,9	35	5,3	Control	
√	**HPAP-041**	RRID:SAMN19776472	F	20,93	24	7,6	T1DM	
√	**HPAP-045**	RRID:SAMN19776476	F	26,2	27	5,2	Control	
√	**HPAP-055**	RRID:SAMN19776485	M	27,9	24	10,4	T1DM	
√	**HPAP-064**	RRID:SAMN19842593	M	16,98	24	13	T1DM	
√	**HPAP-071**	RRID:SAMN19842600	F	15,42	12	9,8	T1DM	
√	**HPAP-072**	RRID:SAMN19842601	M	23,1	19	5,6	Control	
√	**HPAP-094**	RRID:SAMN22562805	F	37,84	38	5	T1DM	
√	**HPAP-098**	RRID:SAMN22562809	F	34,9	30	11	T1DM	
√	**HPAP-113**	RRID:SAMN28088751	M	15,92	9	11,1	T1DM	
√	**HPAP-114**	RRID:SAMN28088752	F	25,66	21	5,3	Control	
√	**HPAP-122**	RRID:SAMN29419888	F	30,05	21	5,5	Control	
×	**HPAP-042**	RRID:SAMN19776473	M	17,9	1	5,6	Control	missing age-matched child with T1D
×	**HPAP-044**	RRID:SAMN19776475	F	12	3	5,3	Control	missing age-matched child with T1D
×	**HPAP-073**	RRID:SAMN19842602	M	25,92	24	8,2	T1DM	data disturbance in CD4+CD8low population
×	**HPAP-131**	RRID:SAMN31536832	M	22,5	23	5,1	Control	data disturbance in CD4+CD8low population
×	**HPAP-135**	RRID:SAMN32643783	M	26,35	18	14,7	T1DM	data disturbance in CD4+CD8low population

Supplementary Table 7. List of flow cytometry samples from the HPAP database that were used in this study.
