# Supplementary material for: Imbalance of stem-like and effector T cell states in children with early type 1 diabetes across conventional and regulatory subsets
Source: Nat Commun. 2025 Dec 11;16:11301. doi: 10.1038/s41467-025-66459-4 (PMC12722300; doi:10.1038/s41467-025-66459-4)
Supplement: Supplementary file 2 — Reporting Summary [file 41467_2025_66459_MOESM2_ESM.pdf]

nature portfolio

Corresponding author(s): Ondrej Stepanek

Last updated by author(s): Oct 10, 2025

Reporting Summary

Nature Portfolio wishes to improve the reproducibility of the work that we publish. This form provides structure for consistency and transparency in reporting. For further information on Nature Portfolio policies, see our Editorial Policies and the Editorial Policy Checklist.

Statistics

For all statistical analyses, confirm that the following items are present in the figure legend, table legend, main text, or Methods section.

n/a	Confirmed	
☐	☒	The exact sample size (*n*) for each experimental group/condition, given as a discrete number and unit of measurement
☐	☒	A statement on whether measurements were taken from distinct samples or whether the same sample was measured repeatedly
☐	☒	The statistical test(s) used AND whether they are one- or two-sided<br>*Only common tests should be described solely by name; describe more complex techniques in the Methods section.*
☐	☒	A description of all covariates tested
☐	☒	A description of any assumptions or corrections, such as tests of normality and adjustment for multiple comparisons
☐	☒	A full description of the statistical parameters including central tendency (e.g. means) or other basic estimates (e.g. regression coefficient) AND variation (e.g. standard deviation) or associated estimates of uncertainty (e.g. confidence intervals)
☐	☒	For null hypothesis testing, the test statistic (e.g. *F*, *t*, *r*) with confidence intervals, effect sizes, degrees of freedom and *P* value noted<br>*Give P values as exact values whenever suitable.*
☐	☒	For Bayesian analysis, information on the choice of priors and Markov chain Monte Carlo settings
☒	☐	For hierarchical and complex designs, identification of the appropriate level for tests and full reporting of outcomes
☐	☒	Estimates of effect sizes (e.g. Cohen's *d*, Pearson's *r*), indicating how they were calculated

Our web collection on statistics for biologists contains articles on many of the points above.

Software and code

Policy information about availability of computer code

Data collection	SpectroFlo 3.1.0 (Cytek)<br>NovaSeq Control Software, v. 1.8.1
Data analysis	FlowJo software v10.10 + FlowJo plugin FlowAI<br>Cell Ranger 5.0.1<br>R 4.2.1.<br>IDEIS - https://doi.org/10.3389/fimmu.2024.1446931<br>Own scripts available at GITHUB: https://github.com/Lab-of-Adaptive-Immunity/dia

For manuscripts utilizing custom algorithms or software that are central to the research but not yet described in published literature, software must be made available to editors and reviewers. We strongly encourage code deposition in a community repository (e.g. GitHub). See the Nature Portfolio guidelines for submitting code & software for further information.

Data

Policy information about availability of data

All manuscripts must include a data availability statement. This statement should provide the following information, where applicable:

- Accession codes, unique identifiers, or web links for publicly available datasets
- A description of any restrictions on data availability
- For clinical datasets or third party data, please ensure that the statement adheres to our policy

The scRNAseq data and flow cytometry data generated in this study have been deposited on Zenodo under DOI 10.5281/zenodo.17280189 [10.5281/zenodo.17280189]. The raw sequencing data are protected and are not available due to data privacy laws. Data from previously published studies used for validation of our findings are available in the Gene Expression Omnibus (GEO) database under the following accession codes: GSE237218 [https://www.ncbi.nlm.nih.gov/geo/query/acc.cgi?acc=GSE237218], GSE123658 [https://www.ncbi.nlm.nih.gov/geo/query/acc.cgi?acc=GSE123658], GSE10586 [https://www.ncbi.nlm.nih.gov/geo/query/acc.cgi?acc=GSE10586], GSE221297 [https://www.ncbi.nlm.nih.gov/geo/query/acc.cgi?acc=GSE221297], in the European Genome-phenome Archive (EGA) under the following accession codes: EGAD00001005767 [https://ega-archive.org/datasets/EGAD00001005767], EGAD00001005768 [https://ega-archive.org/datasets/EGAD00001005768], and in the Synapse database under the accession code syn53641849 [https://www.synapse.org/Synapse:syn53641849].

All display items presented in the main manuscript and supplementary information can be reproduced from data and code that are available in public repositories. The raw numbers for charts and graphs are available in the Source Data file whenever possible. Source data are provided with this paper.

Research involving human participants, their data, or biological material

Policy information about studies with human participants or human data. See also policy information about sex, gender (identity/presentation), and sexual orientation and race, ethnicity and racism.

Reporting on sex and gender	We report on the sex of the subjects. We did not collect information on the gender.
Reporting on race, ethnicity, or other socially relevant groupings	These data were not collected.
Population characteristics	The age group (not the age itself to prevent the identification of subjects) and sex are indicated in Figure 1A.
Recruitment	T1D participants were recruited purely based on the diabetes diagnosis and their consent. Healthy donors were recruited by directly contacting parents and based on their consent.
Ethics oversight	Ethics Committee of the Motol University Hospital, Prague, Czech Republic

Note that full information on the approval of the study protocol must also be provided in the manuscript.

Field-specific reporting

Please select the one below that is the best fit for your research. If you are not sure, read the appropriate sections before making your selection.

☒ Life sciences ☐ Behavioural & social sciences ☐ Ecological, evolutionary & environmental sciences

For a reference copy of the document with all sections, see nature.com/documents/nr-reporting-summary-flat.pdf

Life sciences study design

All studies must disclose on these points even when the disclosure is negative.

Sample size	The sample size of 30 T1D donors was determined before the start of the experiments to achieve a balance between the costs of the experiments on the one hand and the number of cells per donor and the sufficient number of donors for statistical analysis on the other hand. The number of healthy donors was planned to match the number of T1D donors, but we were not able to recruit the target number because the samples were collected in the middle of the COVID19 pandemic. At the time of sample collection, no scRNA-seq datasets and very few robust bulk transcriptomic studies on T1D were available. Consequently, it was not possible to reliably estimate variability or effect size a priori, and we did not perform a formal power calculation.
Data exclusions	One healthy donor was excluded based on poor viability of cells after thawing observed by flow cytometry and by subsequent scRNAseq analysis. This indicated an issue with the freezing of this particular sample. Exceptionally poor viability was a pre-established qualitative exclusion criterium.
Replication	The reproducibility is based on treating the particular donors as biological replicates. Selected results from the scRNAseq analysis were replicated using flow cytometry. Selected results were replicated using publicly available scRNAseq, bulk RNAseq, and/or flow cytometry data from independent cohorts.
Randomization	No experimental groups were made.
Blinding	No blinding was performed. The processing of the samples included standard freezing protocol, thawing, and staining of the samples and eventual sorting and processing for scRNAseq using standardized protocols. The cells were sorted into shared tubes (pooling) and the pools of

healthy and T1D donors were processed together. The processing of the scRNAseq data up to the point of the final quantification was identical for all samples.

Reporting for specific materials, systems and methods

We require information from authors about some types of materials, experimental systems and methods used in many studies. Here, indicate whether each material, system or method listed is relevant to your study. If you are not sure if a list item applies to your research, read the appropriate section before selecting a response.

Materials & experimental systems

n/a	Involved in the study
☐	☒ Antibodies
☒	☐ Eukaryotic cell lines
☒	☐ Palaeontology and archaeology
☒	☐ Animals and other organisms
☒	☐ Clinical data
☒	☐ Dual use research of concern
☒	☐ Plants

Methods

n/a	Involved in the study
☒	☐ ChIP-seq
☐	☒ Flow cytometry
☒	☐ MRI-based neuroimaging

Antibodies

Antibodies used

The following antibodies were used for flow cytometry staining: anti-CD3 (clone MEM-57, Exbio #A7-202-T100), anti-CD3 (clone UCHT1, BD #561007), anti-CD4 (clone SK3, Biolegend #344632), anti-CD4 (clone OKT4, Biolegend #317433), anti-CD16 (clone B73.1, Biolegend #360729), anti-CD8 (clone MEM-31, Exbio #1A-207-T100), anti-CD25 (clone M-A251, Biolegend #356146), anti-CD45RA (clone MEM-56, Exbio #1F-223-T100), anti-CD45RO (clone UCHL1, Biolegend #304232), anti-CD45R/B220 (clone RA3-6B2, Biolegend #103248), anti-CD56 APC-R700 (clone NCAM16.2, BD #565140), anti-CD127 (clone A019D5, Biolegend #351333), anti-CD137 (clone 4B4, eBioscience #25-1379-42), anti-CD183 (clone G025H7, Biolegend #353705), anti-CD184 (clone 12G5, Exbio #1P-146-T100), anti-CD196 (clone G034E3, Biolegend #353429), anti-CD197 (clone G043H7, Biolegend #353227), anti-CD226 (clone 11A8, Exbio #1P-926-T100), anti-EOMES (clone X4-83, BD Pharmingen #567167), anti-FOXP3 (clone 206D, Biolegend #320125), anti-IκBα (clone L35A5, Cell Signaling #5743S), anti-Granzyme B (clone QA16A02, Biolegend #372213), anti-Granzyme K (clone GM26E7, Biolegend #370513), anti-HLA-DR (clone LN3, Biolegend #327019), anti-Ki-67 (clone Ki-67, Biolegend #350535), anti-TCR gamma/delta (clone 11F2, Exbio #T7-912-T100), anti-TCR Vα24-Jα18 (clone 6B11, Biolegend #342929).
For sorting, cells were incubated on ice for 5 minutes with Human TrueStain FcX (BioLegend #422301) and for additional 30 minutes with anti-human CD4 and CD8 antibodies (CD8 APC, LT8, Exbio #1A-817-T100; CD4 AF700, MEM-241, Exbio # A7-539-T100) and one of the hashtag antibodies (TotalSeq™-C0251 anti-human Hashtag 1 Antibody, LNH-94 2M2, BioLegend #394661; TotalSeq™-C0252 anti-human Hashtag 2 Antibody, LNH-94 2M2, BioLegend #394663; TotalSeq™-C0253 anti-human Hashtag 3 Antibody, LNH-94 2M2, BioLegend #394665; TotalSeq™-C0254 anti-human Hashtag 4 Antibody, LNH-94 2M2, BioLegend #394667; TotalSeq™-C0255anti-human Hashtag 5 Antibody, LNH-94 2M2, BioLegend #394669; TotalSeq™-C0256 anti-human Hashtag 6 Antibody, LNH-94 2M2, BioLegend #394671; TotalSeq™-C0257 anti-human Hashtag 7 Antibody, LNH-94 2M2, BioLegend #394673; TotalSeq™-C0258 anti-human Hashtag 8 Antibody, LNH-94 2M2, BioLegend #394675; TotalSeq™-C0259 anti-human Hashtag 9 Antibody, LNH-94 2M2, BioLegend #394677; TotalSeq™-C0260 anti-human Hashtag 10 Antibody, LNH-94 2M2, BioLegend #394679) and with Hoechst 33258 for viability right before sorting.

Validation

The antibody were validated by the manufacturers. The information about the validation of particular antibodies can be found online using their catalogue numbers.

Plants

Seed stocks

Report on the source of all seed stocks or other plant material used. If applicable, state the seed stock centre and catalogue number. If plant specimens were collected from the field, describe the collection location, date and sampling procedures.

Novel plant genotypes

Describe the methods by which all novel plant genotypes were produced. This includes those generated by transgenic approaches, gene editing, chemical/radiation-based mutagenesis and hybridization. For transgenic lines, describe the transformation method, the number of independent lines analyzed and the generation upon which experiments were performed. For gene-edited lines, describe the editor used, the endogenous sequence targeted for editing, the targeting guide RNA sequence (if applicable) and how the editor was applied.

Authentication

Describe any authentication procedures for each seed stock used or novel genotype generated. Describe any experiments used to assess the effect of a mutation and, where applicable, how potential secondary effects (e.g. second site T-DNA insertions, mosiacism, off-target gene editing) were examined.

Flow Cytometry

Plots

Confirm that:

- [x] The axis labels state the marker and fluorochrome used (e.g. CD4-FITC).
- [x] The axis scales are clearly visible. Include numbers along axes only for bottom left plot of group (a 'group' is an analysis of identical markers).
- [x] All plots are contour plots with outliers or pseudocolor plots.
- [x] A numerical value for number of cells or percentage (with statistics) is provided.

Methodology

Sample preparation

The samples were obtained from 30 children with T1D at T0 and from 29 of these children at T1. One T1D participant withdrew from the study before the one-year follow-up visit. Their sample was included in all analysis except for paired statistical testing. A cohort of 13 healthy controls was also enrolled. The parents/caregivers and the participants signed a written consent.

Three to ten mL of peripheral blood were collected into EDTA-coated tubes, kept on ice, and transferred from Motol University Hospital to the Institute of Molecular Genetics within two hours. PBMCs were separated using Ficoll-Paque (GE Healthcare) and immediately frozen following a cryopreservation protocol (10x Genomics). Briefly, 4 ml of Ficoll-Paque was overlaid with blood and centrifuged at 400g for 30 minutes at room temperature (brake set to one). The mononuclear cell layer was washed in PBS and resuspended in RPMI medium containing 40% FBS. After adding an equal volume of freezing medium (RPMI, 40% FBS, and 30% DMSO), two to five aliquots of PBMCs were frozen and stored in liquid nitrogen. PBMCs were gently thawed by slow, sequential dilution in RPMI medium containing 10% FBS. To minimize cell stress, wide-bore tips were used during the thawing process.

Flow cytometry was performed in two batches. In each batch, aliquoted PBMCs stored in the liquid nitrogen were gently thawed by slow, sequential dilution in RPMI medium containing 10% FBS. Cells were counted and the same amount of cells (1.2 million in batch one, 2 million in batch two) were used for staining. The counted cells were divided into three equal parts. The first part was used for extracellular staining of live cells, the other two parts were used for intracellular staining of fixed cells. To prevent unspecific binding to Fc receptors, Human TrueStain FcX (BioLegend #422301) was added to all staining mixes. For staining of extracellular markers (Panel 1: CD4 OKT4 BV421, CD16 BV510, TCR Vα24-Jα18 BV605, CD45RO BV650, CD197 BV711, CD45RA FITC, CD3 UCHT1 PE-Cy5, HLA-DR PerCP/Cy5.5, CD183 PE, CD25 PE/Fire700, CD196 PE/Dazzle 594, TCR gamma/delta PE-Cy7, CD8 APC, CD56 APC-R700), cells were incubated with diluted antibodies and LIVE/DEAD Fixable Near-IR Dead Cell Stain Kit (Invitrogen #L34975) for 30 min on ice immediately after isolation. For staining of intracellular markers and transcription factors, cells were first stained for 30 min on ice with the mix containing LIVE/DEAD Fixable Near-IR Dead Cell Stain Kit (Invitrogen #L34975) and extracellular markers (CD3 MEM-57 AF700, CD4 SK3 BV421, CD8 APC, CD25 PE/Fire700, CD45RA FITC, CD45RO BV650, CD45R/B220 BV510) and then fixed and permeabilized using the eBioscience Foxp3 / Transcription Factor Staining Buffer Set (Invitrogen #00-5523-00) according to the manufacturer’s instructions, washed, and stained overnight with antibodies for intracellular markers in 4°C (Panel 2: extracellular markers and CD127 BV605, Ki-67 BV750, HLA-DR PerCP/Cy5.5, CD226 PE, FOXP3 PE/Dazzle 594, CD137 Pe/Cy7; Panel 3: extracellular markers and CD127 BV605, Ki-67 BV750, IκBα AF488, GZMK PerCP/Cy5.5, CD184 PE, Eomes PE/CF594, GZMB PE/Cy7).

Instrument

Cytek Aurora flow cytometer, configuration 4L 16V-14B-10YG-8R (Cytek)

Software

FlowJo software v10.10 (BD Life Sciences)

Cell population abundance

The sorted cells were analyzed by scRNAseq which revealed individual cell subtypes. For this reason, the post-sort purity was not determined in the final experiments. When protocol was optimized, the purity was tested.

Gating strategy

Gating strategies are indicated in the Supplementary Information

- [x] Tick this box to confirm that a figure exemplifying the gating strategy is provided in the Supplementary Information.
